# Abstracts from the NIHR INVOLVE Conference 2017: London, UK. 28 November 2017

**DOI:** 10.1186/s40900-017-0075-x

**Published:** 2017-11-28

**Authors:** Delia Muir, Lidewij Eva Vat, Malori Keller, Tim Bell, Clara R. Jørgensen, Nanna B. Eskildsen, Anna T. Johnsen, Raksha Pandya-Wood, Steven Blackburn, Ruth Day, Carol Ingram, Julie Hapeshi, Samaira Khan, Delia Muir, Wendy Baird, Sue H. Pavitt, Richard Boards, Janet Briggs, Ellen Loughhead, Mariya Patel, Rameesa Khalil, David Cooper, Peter Day, Jenny Boards, Jianhua Wu, Timothy Zoltie, Sophy Barber, Wendy Thompson, Kate Kenny, Jenny Owen, Martin Ramsdale, Kara Grey-Borrows, Nigel Townsend, Judith Johnston, Katie Maddison, Harry Duff-Walker, Katie Mahon, Lily Craig, Rebecca Collins, Alice O’Grady, Sarah Wadd, Adrian Kelly, Maureen Dutton, Michelle McCann, Rebecca Jones, Elspeth Mathie, Helena Wythe, Diane Munday, Paul Millac, Graham Rhodes, Nick Roberts, Jean Simpson, Nat Barden, Penny Vicary, Amander Wellings, Fiona Poland, Julia Jones, Jahanara Miah, Howard Bamforth, Anna Charalambous, Piers Dawes, Steven Edwards, Iracema Leroi, Valeria Manera, Suzanne Parsons, Ruth Sayers, Vanessa Pinfold, Paul Dawson, Bliss Gibbons, John Gibson, Charley Hobson-Merrett, Catherine McCabe, Tim Rawcliffe, Lucy Frith, Bernard Gudgin, Amander Wellings, Adele Horobin, Colleen Ewart, Fred Higton, Stevie Vanhegan, Raksha Pandya-Wood, Jane Stewart, Andy Wragg, Paula Wray, Kirsty Widdowson, Lisa Jane Brighton, Sophie Pask, Hamid Benalia, Sylvia Bailey, Marion Sumerfield, Simon Etkind, Fliss E. M. Murtagh, Jonathan Koffman, Catherine J. Evans, Susan Hrisos, Julie Marshall, Lyndsay Yarde, Bren Riley, Paul Whitlock, Jacqui Jobson, Safia Ahmed, Judith Rankin, Lydia Michie, Jason Scott, Caroline R. Barker, Megan Barlow-Pay, Aisha Kekere-Ekun, Aniqa Mazumder, Aniqa Nishat, Rebecca Petley, Louca-Mai Brady, Lorna Templeton, Erin Walker, Darren Moore, Liz Shaw, Michael Nunns, Jo Thompson Coon, Paula Blomquist, Sarah Cochrane, Natalie Edelman, Josina Calliste, Jackie Cassell, Laura B. Mader, Sabine Kläger, Ian B. Wilkinson, Thomas F. Hiemstra, Mel Hughes, Angela Warren, Peter Atkins, Hazel Eaton, Julia Keenan, Fiona Poland, Helena Wythe, Amander Wellings, Penny Vicary, Carol Rhodes, Magdalena Skrybrant, Steven Blackburn, Lucy Chatwin, Mary-Anne Darby, Andrew Entwistle, Diana Hull, Naimh Quann, Gary Hickey, Krysia Dziedzic, Sabrina A. Eltringham, Jim Gordon, Sue Franklin, Joni Jackson, Nick Leggett, Philippa Davies, Manjula Nugawela, Lauren Scott, Verity Leach, Alison Richards, Anthony Blacker, Paul Abrams, Jitin Sharma, Jenny Donovan, Penny Whiting, Simon R. Stones, Catherine Wright, Kate Boddy, Jenny Irvine, Jim Harris, Neil Joseph, Michele Kok, Andy Gibson, David Evans, Sally Grier, Alasdair MacGowan, Rachel Matthews, Constantina Papoulias, Cherelle Augustine, Maurice Hoffman, Mark Doughty, Heidi Surridge, Doreen Tembo, Amanda Roberts, Eleni Chambers, Daniel Beever, Martin Wildman, Rosemary L. Davies, Sophie Staniszewska, Richard Stephens, Sara Schroter, Amy Price, Tessa Richards, Andrew Demaine, Rebecca Harmston, Jim Elliot, Ella Flemyng, Lise Sproson, Liz Pryde, Heath Reed, Gill Squire, Andy Stanton, Joe Langley, Moya Briggs, Philip Brindle, Rod Sanders, Christopher McDermott, Coyle David, Heron Nicola, Davies Simon, Wilkie Martin, Tina Coldham, Claire Ballinger, Lynn Kerridge, Mark Mullee, Caroline Eyles, Megan Barlow-Pay, Gary Hickey, Tracey Johns, Jon Paylor, Katie Turner, Lisa Whiting, Sheila Roberts, Julia Petty, Gary Meager, Anna Grinbergs-Saull, Natasha Morgan, Kati Turner, Flavia Collins, Sarah Gibson, Siobhan Passmore, Liz Evans, Stuart A. Green, Jenny Trite, Rachel Matthews, Susan Hrisos, Richard Thomson, Dave Green, Helen Atkinson, Alex Mitchell, Lynne Corner, Anne Mc Kenzie AM, Rebecca Nguyen, Belinda Frank, Ngaire McNeil, Hayley Harrison

**Affiliations:** 1 Leeds Institute of Clinical Trials Research, University of Leeds, Leeds, UK; 2 Newfoundland and Labrador’s Support for People and Patient-Oriented Research and Trials Unit, Memorial University Newfoundland, St. Johns, Newfoundland and Labrador Canada; 3 Saskatchewan Centre for Patient-Oriented Research, Health Quality Council, Saskatoon, Saskatchewan Canada; 4 Canadian Institutes of Health Research, Ottawa, Ontario Canada; 5 Department of Disability, Inclusion and Special Needs, School of Education, University of Birmingham, Birmingham, UK; 6 Department of Palliative Medicine, Bispebjerg Hospital, DK-2400 Copenhagen, Denmark; 7 Department of Psychology, University of Southern Denmark, Odense, Denmark; 8 National Institute for Health Research, Research Design Service East Midlands, Department of Health Sciences, University of Leicester, Leicester, UK; 9 National Institute for Health Research, Research Design Service West Midlands, Research Institute for Primary Care & Health Sciences, Keele University, Keele, UK; 10 Public contributor involved with the National Institute for Health Research, Research Design Service Public Involvement Community, Derby, UK; 11 National Institute for Health Research, Research Design Service South West, Gloucestershire Royal Hospital, Gloucester, UK; 12 National Institute for Health Research, Research Design Service Yorkshire and Humber, School of Health and Related Research, University of Sheffield, Sheffield, UK; 13 National Institute for Health Research, Research Design Service Yorkshire and Humber, Leeds Institute for Clinical Trials Research, University of Leeds, Leeds, UK; 14 School of Dentistry, University of Leeds, Leeds, UK; 15 The SMILE AIDERS Patient Public Involvement & Engagement Forum School of Dentistry, University of Leeds, Leeds, UK; 16 Batley Girls High School, Batley, UK; 17 Theatre of Debate, London, UK; 18 School of Performance & Cultural Industries, University of Leeds, Leeds, UK; 19 Substance Misuse and Ageing Research Team (SMART), Institute of Applied Social Research, University of Bedfordshire, Luton, UK; 20 CRIPACC, University of Hertfordshire, Hatfield, Hertfordshire, UK; 21 Public Involvement in Research Group, University of Hertfordshire, Hatfield, Hertfordshire, UK; 22 INsPIRE PPI Group, Cambridgeshire Community Services NHS Trust, Ely, Cambridgeshire, UK; 23 Cambridge University Hospital (CUH) Patient and Public Involvement Panel, Cambridgeshire, UK; 24 Service User and Research Group, Cambridge and Peterborough Foundation Trust, Cambridgeshire, UK; 25 Public & Patient Involvement in Research (PPIRes), Norfolk and Suffolk, UK; 26 University of East Anglia, Norwich, Norfolk, UK; 27 Division of Neuroscience and Experimental Psychology, University of Manchester, Manchester, UK; 28 SENSE-Cog Research User Group, Division of Neuroscience and Experimental Psychology, University of Manchester, Manchester, UK; 29 Department of Health Sciences, European University Cyprus, Nicosia, Cyprus; 30 Manchester Centre for Audiology and Deafness (ManCAD), Manchester Academic Health Science Centre, University of Manchester, Manchester, UK; 31 Public Programmes Team, Research and Innovation Division, Central Manchester University Hospitals NHS Foundation Trust, Manchester, UK; 32 CoBTeK COgnition Behaviour Technology, Universite de Nice Sophia Antipolis, Nice, France; 33 The McPin Foundation, London, UK; 34 Lancashire Care NHS Foundation Trust, Preston, UK; 35 Plymouth University Peninsula Schools of Medicine, Plymouth, UK; 36 University of Birmingham, Birmingham, UK; 37 National Institute for Health Research (NIHR), Research Design Service North West, University of Liverpool, Liverpool, UK; 38 Public representative, Thames Valley, UK; 39 Public representative, Norfolk, UK; 40 National Institute for Health Research (NIHR) Nottingham Biomedical Research Centre, Nottingham, UK; 41 Nottingham University Hospitals NHS Trust, Queens Medical Centre, Nottingham, UK; 42 Public representative, Nottingham, UK; 43 National Institute for Health Research (NIHR) East Midlands Research Design Service, Department of Health Sciences, University of Leicester, Leicester, UK; 44 National Institute for Health Research (NIHR) East Midlands Research Design Service, School of Medicine, University of Nottingham, Nottingham Health Science Partners, Queen’s Medical Centre, Nottingham, UK; 45 National Institute for Health Research (NIHR) Nottingham Biomedical Research Centre, Queen’s Medical Centre, Nottingham, UK; 46 INVOLVE Coordinating Centre, University of Southampton, Southampton, UK; 47 Department of Palliative Care, Policy and Rehabilitation, Cicely Saunders Institute, King’s College London, London, UK; 48 Wolfson Palliative Care Research Centre, Hull York Medical School, University of Hull, Hull, UK; 49 Department of Palliative Medicine, Sussex Community NHS Foundation Trust, Brighton, UK; 50 Institute of Health & Society, Newcastle University, Newcastle upon Tyne, UK; 51 InvolveNorthEast, Newcastle upon Tyne, UK; 52 Healthwatch Newcastle, Newcastle upon Tyne, UK; 53 Riverside Project, Newcastle upon Tyne, UK; 54 Advocacy Centre North, Newcastle upon Tyne, UK; 55 Health and Race Equality Forum, Newcastle upon Tyne, UK; 56 National Institute for Health Research Southampton Clinical Research Facility and Biomedical Research Centre, University Hospital Southampton NHS Foundation Trust, Southampton, Hampshire, UK; 57 National Institute for Health Research Design Service South Central, University of Southampton, Southampton, Hampshire, UK; 58 Young Adult Patient and Public Involvement Group member, Southampton, Hampshire, UK; 59 Kingston and St George’s Joint Faculty and Independent Research Consultant, London, UK; 60 Independent Research Consultant, Bristol, UK; 61 Centre for Outcomes and Experiences Research in Child Health, Illness and Disease, Great Ormond Street Hospital for Children NHS Foundation Trust, London, UK; 62 National Institute of Health Research Peninsula Collaboration for Leadership in Applied Health Research & Care, University of Exeter Medical School, Exeter, UK; 63 Public Health England, London, UK; 64 National Institute for Health Research (NIHR) Health Protection Research Unit in Blood Borne and Sexually Transmitted Infections at UCL, London, UK; 65 University Hospitals Bristol NHS Foundation Trust, Bristol, UK; 66 Brighton & Sussex Medical School, Brighton, UK; 67 University of Brighton, Brighton, UK; 68 School of Clinical Medicine, University of Cambridge, Cambridge, UK; 69 Cambridge Clinical Trials Unit, Cambridge University Hospitals NHS Foundation Trust, Cambridge, UK; 70 Faculty of Health and Social Sciences, Bournemouth University, Bournemouth, UK; 71 PIER (Public Involvement in Education and Research) partnership, Bournemouth University, Bournemouth, UK; 72 Research and Development, Dorset Healthcare University NHS Foundation Trust, Dorset, UK; 73 University of East Anglia, Norwich, Norfolk, UK; 74 University of Hertfordshire, Hatfield, Hertfordshire, UK; 75 Members of the Patient and Public in Research Group (PPIRes), NHS South Norfolk Clinical Commissioning Group, Norwich, Norfolk, UK; 76 NIHR Research Design Service West Midlands, Research Institute for Primary Care & Health Sciences, Keele University, Staffordshire, UK; 77 NIHR Collaboration for Leadership in Health Research and Care West Midlands, Institute of Applied Health Research, University of Birmingham, Birmingham, UK; 78 Academic Health Science Network West Midlands, Institute of Translational Medicine, Queen Elizabeth Hospital, Birmingham, UK; 79 NIHR Clinical Research Network West Midlands, Greyfriars Business Park, Stafford, UK; 80 NIHR/Wellcome Trust Birmingham Clinical Research Facility, University Hospitals Birmingham NHS Foundation Trust, Birmingham, UK; 81 INVOLVE, University of Southampton Science Park, Southampton, UK; 82 Directorate of Therapeutics and Palliative, Sheffield Teaching Hospitals NHS Foundation Trust, Sheffield, UK; 83 The National Institute for Health Research Collaboration for Leadership in Applied Health Research and Care West (NIHR CLAHRC West) at University Hospitals Bristol NHS Foundation Trust, Bristol, UK; 84 Population Health Sciences, Bristol Medical School, University of Bristol, Bristol, UK; 85 University Hospitals Coventry and Warwickshire, Coventry, UK; 86 North Bristol Trust, Bristol, UK; 87 NIHR: CRN Children/Arthritis Research UK Paediatric Rheumatology Clinical Studies Group, Liverpool, UK; 88 NIHR CLAHRC South West Peninsula (PenCLAHRC), University of Exeter Medical School, Exeter, UK; 89 NIHR CLAHRC North West Coast (CLAHRC NWC), Based at Division of Health Research, Lancaster University, Lancaster, UK; 90 Peninsula Public Involvement Group (PenPIG), PenCLAHRC, South West Peninsula, Exeter, UK; 91 Public Reference Panel (PRP), CLAHRC NWC, North West Coast area, Liverpool, UK; 92 Department of Health and Social Sciences, University of the West of England, Bristol, UK; 93 Department of Medical Microbiology, North Bristol NHS Trust, Bristol, UK; 94 NIHR CLAHRC Northwest London, Imperial College London/Chelsea and Westminster NHS Foundation Trust, London, UK; 95 NIHR CLAHRC South London, King’s College, London, UK; 96 The King’s Fund, London, UK; 97 NIHR Evaluation Trials and Studies Coordinating Centre (NETSCC), Southampton, UK; 98 Public member, NETSCC Public Involvement Virtual Network and Public member of a Trial Steering Committee, Southampton, UK; 99 Public member, NETSCC PPI Reference Group, Southampton, UK; 100 Clinical Trials Research Unit, School of Health and Related Research, University of Sheffield, Sheffield, South Yorkshire UK; 101 Sheffield Teaching Hospitals NHS Foundation Trust, Sheffield, South Yorkshire UK; 102 Department of Health and Social Sciences, University of the West of England, Bristol, UK; 103 National Institute for Health Research, Collaborations for Leadership in Applied Health Research and Care West (NIHR CLAHRC West), University Hospitals Bristol NHS Foundation Trust, Bristol, UK; 104 Warwick Research in Nursing, Warwick Medical School, University of Warwick, Warwick, UK; 105 Involved and engaged patient and carer, London, UK; 106 The BMJ, London, UK; 107 Department of Continuing Education, University of Oxford, Oxford, UK; 108 Involved and engaged patient, London, UK; 109 Health Research Authority, London, UK; 110 BioMed Central, London, UK; 111 NIHR Devices for Dignity Health Technology Co-operative, Sheffield, UK; 112 Lab4Living, Art and Design Research Centre, Sheffield Hallam University, Sheffield, UK; 113 Sheffield Institute for Translational Neuroscience, University of Sheffield, Sheffield, UK; 114 NIHR Devices for Dignity Healthcare Technology Co-operative at Sheffield Teaching Hospitals NHS Foundation Trust, Royal Hallamshire Hospital, Glossop Road, Sheffield, S10 2JF UK; 115 Institute for Applied Clinical Sciences, Keele University, Keele, Staffordshire UK; 116 University Hospital of North Midlands, Newcastle Rd, Stoke-on-Trent, Staffordshire, ST46QG UK; 117 Renal Medicine, Sheffield Teaching Hospitals NHS Foundation Trust, Sheffield, UK; 118 Wessex PIN, Southampton, UK; 119 NETSCC, University of Southampton, Southampton, UK; 120 Research Design Service South Central, Southampton General Hospital, Southampton, UK; 121 NIHR INVOLVE, University of Southampton, Southampton, UK; 122 NIHR Research Design Service East of England, University of Essex, Essex, UK; 123 NIHR Research Design Service London, Kings College London, London, UK; 124 Population Health Research Institute, St George’s, University of London, London, UK; 125 Department of Nursing and Social Work, University of Hertfordshire, Hatfield, Hertfordshire, England; 126 Alzheimer’s Society, London, UK; 127 Population Health Research Institute, St George’s, University of London, London, UK; 128 NIHR CLAHRC Northwest London, Imperial College London/Chelsea and Westminster NHS Foundation Trust, London, UK; 129 Central and Northwest London NHS Foundation Trust, London, UK; 130 Institute of Health & Society, Newcastle University, Newcastle, UK; 131 Faculty of Medical Sciences Engage, Newcastle University, Newcastle, UK; 132 Consumer and Community Health Research Network, Perth, Australia; 133 Telethon Kids Institute, Perth, Australia

## O2 Learning from Other Fields: can arts based approaches improve the diversity of involvement?

### Delia Muir (d.p.muir@leeds.ac.uk)

#### Leeds Institute of Clinical Trials Research, University of Leeds, Leeds, UK

**Aims**

During this presentation we will share learning from a Wellcome Trust Engagement Fellowship. We will present examples of arts-based public involvement activities, including a sculpture project with young people and a play about dementia. We aim to raise awareness of what public involvement can gain from the arts; stimulate discussion about the pros and cons of different approaches; and discuss how to encourage more creativity within public involvement.

**Why is it important and to whom?**

Public involvement has been criticised for a lack of diversity and inclusivity. By diversifying the involvement activities which we offer, we may attract a wider variety of people. Arts based activities also have the potential to facilitate discussion in an accessible, safe and fun way. This session may be of particular interest to people who are planning or facilitating public involvement activities (members of the public and researchers).

**What difference has, or could, this project make?**

Throughout the project, both researchers and members of the public have found arts activities stimulating and useful. However people have encountered some practical challenges when running these projects. Specifically, people do not feel they have the necessary skills to plan and facilitate arts activities. I will discuss how we might address that skills gap and invite the audience to suggest what support is needed.

**What will people take away from session?**

- An understanding of what arts/health collaborations can offer public involvement
- Access to resources and contacts to support future projects

**Acknowledgments**

This work is funded by the Wellcome Trust

## O3 Your ticket to co-building in Canada: creating a Patient-Oriented Research course

### Lidewij Eva Vat^1^, Malori Keller^2^, Tim Bell^3^

#### ^1^Newfoundland and Labrador's Support for People and Patient-Oriented Research and Trials Unit, Memorial University Newfoundland, St. Johns, Newfoundland and Labrador, Canada; ^2^Saskatchewan Centre for Patient-Oriented Research, Health Quality Council, Saskatoon, Saskatchewan, Canada; ^3^Canadian Institutes of Health Research, Ottawa, Ontario, Canada

##### **Correspondence:** Lidewij Eva Vat (eva.vat@med.mun.ca)

**How do you change the way people do research?**

In Canada, the *Strategy for Patient-Oriented Research (SPOR)* involves asking people to do research in a different way [1]. The idea of research being done by and with patients isn’t new. Growing our ability to work together is still a challenge, especially across 13 very different provinces and territories.

To meet this challenge, a group of patients, education experts and others from across the country joined up to teach people about doing research together. They created a course that talks about a number of topics. Such as: introduction to health research, meaningful roles patients can play and how to work together as a team. Twenty-eight facilitators – including 12 people with lived experience – were trained and taught the course across Canada. Patients, researchers, health care professionals and others participated alongside one another. They built partnerships, improved their skills and gained more knowledge about working together in research.

Join us on a trip across Canada to:

- Learn how we developed and evaluated the course;
- Learn how the course helps to spread awareness and change the way people do research;
- Hear from facilitators (including a patient) about their experience delivering the course;
- Share experiences and training resources with other participants.

**References**

[1] Canadian Institutes of Health Research. Strategy for Patient-Oriented Research. http://www.cihr-irsc.gc.ca/e/41204.html Accessed 31 August 2017.

## O4 User-involvement in a Danish research project on empowerment of cancer patients in follow up

### Clara R. Jørgensen^1^, Nanna B. Eskildsen^2,3^, Anna T. Johnsen^2,3^

#### ^1^Department of Disability, Inclusion and Special Needs, School of Education, University of Birmingham, Birmingham, UK; ^2^Department of Palliative Medicine, Bispebjerg Hospital, DK-2400 Copenhagen, Denmark; ^3^Department of Psychology, University of Southern Denmark, Odense, Denmark

##### **Correspondence:** Clara R. Jørgensen (c.joergensen@bham.ac.uk)

This presentation will discuss the involvement of former and current cancer patients in a Danish research project on patient empowerment of cancer patients in follow up (2015-). The project has involved a total of 17 patients as advisors, co-researchers and peer interviewers.

User-involvement is a relatively new phenomenon in Denmark and the project is one of the first to incorporate user-involvement into the research process from its beginning.

Documenting involvement in the project’s development and delivery is therefore of key importance both nationally, where it provides an early example of good practice, and internationally, as important lessons can be learned from comparing countries. In the development, design and delivery of user-involvement, the project drew on expertise from the University of Warwick (UK), where user-involvement through UNTRAP (University/User Teaching and Research Action Partnership) has been part of research practice for over 10 years.

The presentation outlines the involvement of service users in the different stages of the research. Challenges and benefits of involving service users in the project will be discussed in relation to the specific Danish context and the particular area of cancer research. In addition, the presentation will reflect on some of the differences and similarities between the UK and Denmark in terms of involvement, suggesting that the experiences and expectations of service users may vary depending on the local context, and that researchers need to be sensitive to these differences when relying on expertise from another country.

**Acknowledgements**

The Empowerment project is funded by the Danish Cancer Society Award Number: R113- A6922-14-S34.

We wish to thank the project group and the 17 co-researchers.

## O8 Being a lay co-applicant on national peer reviewed research funding grants

### Raksha Pandya-Wood^1^, Steven Blackburn^2^, Ruth Day^1,3^, Carol Ingram^2,3^, Julie Hapeshi^4^, Samaira Khan^5^, Delia Muir^6^, Wendy Baird^5^

#### ^1^National Institute for Health Research, Research Design Service East Midlands, Department of Health Sciences, University of Leicester, Leicester, UK; ^2^National Institute for Health Research, Research Design Service West Midlands, Research Institute for Primary Care & Health Sciences, Keele University, Keele, UK; ^3^Public contributor involved with the National Institute for Health Research, Research Design Service Public Involvement Community, Derby, UK; ^4^National Institute for Health Research, Research Design Service South West, Gloucestershire Royal Hospital, Gloucester, UK; ^5^National Institute for Health Research, Research Design Service Yorkshire and Humber, School of Health and Related Research, University of Sheffield, Sheffield, UK; ^6^National Institute for Health Research, Research Design Service Yorkshire and Humber, Leeds Institute for Clinical Trials Research, University of Leeds, Leeds, UK

##### **Correspondence:** Raksha Pandya-Wood (rp185@le.ac.uk)

**Aims of the session**

1. Enabling a better understanding about the role of lay co-applicants throughout research studies and within a research team (considering finance, ethics, legalities, social and access issues).
2. Considering when having a lay co-applicant is appropriate (or not appropriate), including considerations of who/how many.
3. Reflecting on the expectations different research funding streams have regarding lay co- applicants.
4. Considering the experiences (good and bad) of PI members who have been lay co- applicants.
5. Outlining the practical steps to support lay co-applicants.

**Why is it important and to whom?**

Research funders increasingly expect lay co-applicants on national peer reviewed research grant applications, as part of the Public Involvement (PI) in the study. However there is no guidance for either PI members or researchers on the role of being a lay co-applicant, when one is needed or how to support lay co-applicants during the development and delivery of research studies.

**What difference has, or could, this project make?**

This interactive workshop will explore the role, responsibilities and support of lay co- applicants on national peer reviewed research funding grants. The workshop will act as a springboard helping to nationally develop a lay role description and information pack about what it means to be a lay co-applicant.

**What will people take away from your session?**

This co-presented workshop has the potential to improve our understanding of the lay co- applicants in research by helping to develop a role description of and guidance for lay co- applicant. The workshop will help all stakeholders (researchers, PI members, funders) appreciate the value and importance of lay co-applicants, and allow PI members clarity on their role within research teams

**Acknowledgements**

This workshop is presented on behalf of the NIHR Research Design Service Public Involvement Community.

## O9 “Don’t Smile – A love story with a dental theme”: National award-winning theatre and debate disseminating dental research to at-risk adolescents

### Sue H. Pavitt^1,2^, Richard Boards^2^, Janet Briggs^2^, Ellen Loughhead^2,3^, Mariya Patel^2,3^, Rameesa Khalil^2,3^, David Cooper^3^, Peter Day^1^, Jenny Boards^1,2^, Jianhua Wu^1^, Timothy Zoltie^1^, Sophy Barber^1^, Wendy Thompson^1^, Kate Kenny^1^, Jenny Owen^1^, Martin Ramsdale^1^, Kara Grey-Borrows^1^, Nigel Townsend^4^, Judith Johnston^4^, Katie Maddison^5^, Harry Duff-Walker^5^, Katie Mahon^5^, Lily Craig^5^, Rebecca Collins^5^, Alice O'Grady^5^

#### ^1^School of Dentistry, University of Leeds, Leeds, UK; ^2^The SMILE AIDERS Patient Public Involvement & Engagement Forum School of Dentistry, University of Leeds, Leeds, UK; ^3^Batley Girls High School, Batley, UK; ^4^Theatre of Debate, London, UK; ^5^School of Performance & Cultural Industries, University of Leeds, Leeds, UK

##### **Correspondence:** Sue H. Pavitt (s.pavitt@leeds.ac.uk)

**Background**

In Yorkshire approximately 45% of 12-year-olds have rotten teeth, this is the second-worst UK prevalence and is strongly correlated with social inequality. Whilst largely preventable, reaching those most vulnerable is challenging. “Don’t Smile” is a play inspired to test if using theatre might improve oral health knowledge of disadvantaged adolescents.

**Method**

The play was a co-production created with Theatre of Debate (TOD) working in an ‘artist in resident-style’, with patient/public advisors, adolescents, theatre-practitioners and University student in arts and sciences. The focus of the play was to disseminate dental research on Amelogenesis Imperfecta. It also drew wider parallels to the implications of poor oral health and portrayed dental public health messages. The play was performed in secondary schools in areas of significant social deprivation to at-risk adolescents. At each performance the TOD Facilitator initiated a poll relating to oral health awareness and opinions. Following the play she revisited for a change in response and facilitated a debate on the wider implications of poor oral health, social isolation, bullying and NHS dental access. Questionnaires on understanding and acceptability of the play were completed.

**Results**

Our embedded dental public health message dealing with dental trauma was understood by 100% of our audiences of vulnerable teenagers from areas of worst oral health inequalities. They also rated the play excellent/very good with 95.5% saying they would like to see more plays on aspects of oral health and dentistry.

Don’t Smile is an innovation in dental dissemination that successfully: [1] reached BME vulnerable teenagers in Schools in areas of high oral health inequality/deprivation and raised oral health awareness; [2] enhanced research impact targeting seldom-heard audiences at high-risk; [3] capacity-built Performing Arts Students to work on a Science/Health-related topic. [4] was aspirational with an unexpected outcome that sixth-formers were keen to undertake participatory dental research for their Extended Project Qualification. We have established ‘RAISED In Yorkshire’ (ReseArch In Schools Evaluating Dental health), a pupil-peer recording of oral health behaviour.

**Conclusion**

“Don’t Smile” won the 2016 National Coordinating Centre for Engagement Award for engaging with young people and the University of Leeds Public Engagement in Research Award. A theatre and debate approach is an effective media to disseminate research and inform pupils. We have created a web-based documentary to inform how to undertake a similar dissemination project http://medhealth.leeds.ac.uk/dentistry/cohesion/dontsmile.

**Acknowledgment**

This work was supported by DenTCRU, part of the Leeds NIHR Clinical Research Facility.

## O12 Unreliable and incapable? Exploding myths about employing people with lived experience of addictions to lead research

### Sarah Wadd, Adrian Kelly, Maureen Dutton, Michelle McCann, Rebecca Jones

#### Substance Misuse and Ageing Research Team (SMART), Institute of Applied Social Research, University of Bedfordshire, Luton, UK

##### **Correspondence:** Sarah Wadd (sarah.wadd@beds.ac.uk)

Expert-by-experience led research in the field of addictions is rare. Even amongst some addictions researchers, there is a sense that people with lived experience of addictions are unreliable and incapable of leading and delivering research, perhaps because of stereotypes applied to people with addictions which include being untrustworthy, emotionally unstable and incompetent.

The aim of this presentation is to explode these myths. Half our Substance Misuse and Ageing Research Team is made up of people with lived experience of addictions who are being trained to become independent researchers. Known as Public and Expert by Experience Researchers (PEERs), these researchers are employed by our university and have equal status to traditional researchers.

This session will be of interest to anyone who is considering embedding researchers with lived experience in their research teams and to people with lived experience who are interested in becoming researchers. It will be of particular interest to those researching or with lived experience of conditions that are wrongly associated with being incapable such as those experiencing mental health issues and complex social problems. Our model is breaking down barriers and stereotypes. This will be a joint presentation delivered by a PEER and traditional researcher to describe our model and share our learning.

## O13 Regional working in east of England: co-designing a PPI feedback tool

### Elspeth Mathie^1^, Helena Wythe^1^, Diane Munday^2^, Paul Millac^2^, Graham Rhodes^3^, Nick Roberts^3^, Jean Simpson^4^, Nat Barden^5^, Penny Vicary^6^, Amander Wellings^6^, Fiona Poland^7^, Julia Jones^1^

#### ^1^CRIPACC, University of Hertfordshire, Hatfield, Hertfordshire, UK; ^2^Public Involvement in Research Group, University of Hertfordshire, Hatfield, Hertfordshire, UK; ^3^INsPIRE PPI Group, Cambridgeshire Community Services NHS Trust, Ely, Cambridgeshire, UK; ^4^Cambridge University Hospital (CUH) Patient and Public Involvement Panel, Cambridgeshire, UK; ^5^Service User and Research Group, Cambridge and Peterborough Foundation Trust, Cambridgeshire, UK; ^6^Public & Patient Involvement in Research (PPIRes), Norfolk and Suffolk, UK; ^7^University of East Anglia, Norwich, Norfolk, UK

##### **Correspondence:** Elspeth Mathie (e.j.mathie@herts.ac.uk)

**Background**

The importance of feedback is highlighted in the ‘Values and Principles’ [1] from INVOLVE and included in the current Public Involvement consultation on standards [2]. Patient and Public Involvement (PPI) contributors in the East of England (EoE) regional network flagged up the issue that feedback (from researchers to PPI contributors) was minimal or absent, so we co-designed a study to look at this. PPI contributors talked of spending valuable time commenting on complex issues and continue to volunteer without acknowledgement and thanks. The study aims to improve PPI feedback by co-designing a generic PPI Feedback process which can be adapted for individual PPI groups and activities.

**Methods**

The six regional PPI groups involved in the study include those based within the Research Design Service, Universities, hospitals and NHS Trusts. The study used a survey, interviews and 4 month audit. Over 100 respondents completed the survey distributed by the PPI groups and 23 PPI contributors, researchers and PPI leads were interviewed. Following two stakeholder meetings with researchers, PPI representatives and PPI group leads, local feedback tools were co-designed, implemented and trialled in the PPI groups. A second audit was undertaken by PPI representatives and PPI group leads to ascertain whether satisfaction with feedback had improved. Work is ongoing to identify barriers and facilitators to implementing the local tools and to co-develop the local tools to form a single regional EoE tool or process.

**Results**

The results confirmed the anecdotal evidence; feedback is not routine and very variable. Together, our research team (PPI contributors, leads, researchers) will outline our motivations for this research approach and our Feedback Tools. We will also discuss our results on the variation and frequency of feedback, barriers and enablers.

**Conclusion**

We aim to encourage other PPI groups to work together to improve feedback whilst underlining the importance of managing expectations and simultaneously nurturing relationships. A regional PPI Feedback tool or process is in development which we aim to produce and distribute in different user-formats.

**Acknowledgements**

Study stakeholder and research group; PPI group Leads and PPI groups.

**References**

1. INVOLVE. Public involvement in research: values and principles framework. INVOLVE; Eastleigh. 2015.

2. https://sites.google.com/nihr.ac.uk/pi-standards/home

## O15 Overcoming the challenges of involving older people with dementia, hearing and vision problems in research – sharing learning and future progress

### Jahanara Miah^1,5^, Howard Bamforth^2^, Anna Charalambous^3^, Piers Dawes^4^, Steven Edwards^5^, Iracema Leroi^1^, Valeria Manera^6^, Suzanne Parsons^5^

#### ^1^Division of Neuroscience and Experimental Psychology, University of Manchester, Manchester, UK; ^2^SENSE-Cog Research User Group, Division of Neuroscience and Experimental Psychology, University of Manchester, Manchester, UK; ^3^Department of Health Sciences, European University Cyprus, Nicosia, Cyprus; ^4^Manchester Centre for Audiology and Deafness (ManCAD), Manchester Academic Health Science Centre, University of Manchester, Manchester, UK; ^5^Public Programmes Team, Research and Innovation Division, Central Manchester University Hospitals NHS Foundation Trust, Manchester, UK; ^6^CoBTeK COgnition Behaviour Technology, Universite de Nice Sophia Antipolis, Nice, France

##### **Correspondence:** Jahanara Miah (Jahanara.miah@manchester.ac.uk)

**Background**

Involving older adults with dementia, hearing and vision problems in research has traditionally been considered impractical. We are involving older people with these problems in a multi-site European research programme (SENSE-Cog) via three research user groups (RUGs) in the UK, France and Cyprus. SENSE-Cog explores the combined impact of dementia, hearing and vision problems and will develop new tools and at home support to improve quality of life for people living with dementia, hearing and vision problems.

**Materials and Methods**

Older adults with lived experience of cognitive, vision and hearing problems (n=15) and carers (n=10) were recruited via advertisements in the general community to RUGs at three SENSE-cog research sites in Manchester, Nice, and Nicosia. RUG members received research awareness training (RAT) [1] to increase their understanding of research to support meaningful involvement. We supported RUG members by taking a tailored approach taking into account the cognitive, vision and hearing problems of RUG members.

With respect to cognitive problems, group facilitators ensured the RUG sessions were interactive, broke tasks down into bite sized pieces and provided memory aids in the form of discussion notes. Hearing problems were addressed by using quiet rooms, including visual prompts to reinforce auditory information and having one person to speak at a time. For vision problems, different light settings were used and hand-outs were printed in large easily readable font on yellow paper. Facilitators stood close to and facing the participants and kept still while speaking.

Facilitators gave clear instructions, spoke in a clear and audible tone of voice, checked for understanding and re-capped the main discussion points.

**Results**

Individualised strategies to support RUG members with hearing, vision and cognitive impairments offered views on key design aspects of the SENSE-cog study including usability aspects of an online hearing and vision test, a protocol for a controlled trial of a sensory support intervention for people with dementia and study recruitment materials.

Evaluation of the RAT using a questionnaire survey is on-going to assess RUG member’s acceptability and satisfaction with the training. Focus groups and interviews will discover how RUG members had made use of the knowledge and skills provided by the training in the context of SENSE-Cog’s research programme.

**Conclusions**

Meaningful involvement of people with dementia, hearing and vision problems is feasible provided that a tailored and iterative approach is taken to understand the needs of people with cognitive and sensory problems.

**Acknowledgements**

This review is part of the Work Package 5 of the SENSE-Cog project, which has received funding from the European Union’s Horizon 2020 research and innovation programme under grant agreement no. 668648.

**References**

1. Grundy, A. et al. Evaluation of a co-delivered training package for community mental health professionals on service user and carer involved care planning. Journal of Psychiatric and Mental Health Nursing (2017) DOI: 10.1111/jpm.12378. Publication link: 94ea9901-2d15- 472e-b3f6-bbd9a4d3e560

## O17 Moving from Patient and Public Involvement towards co-production in a large trial for supporting people with ongoing mental health needs in Primary Care: the PARTNERS2 experience

### Ruth Sayers^1^, Vanessa Pinfold^1^, Paul Dawson^2^, Bliss Gibbons^4^, John Gibson^1^, Charley Hobson-Merrett^3^, Catherine McCabe^3^, Tim Rawcliffe^2^

#### ^1^The McPin Foundation, London, UK; ^2^Lancashire Care NHS Foundation Trust, Preston, UK; ^3^Plymouth University Peninsula Schools of Medicine, Plymouth, UK; ^4^University of Birmingham, Birmingham, UK

##### **Correspondence:** Vanessa Pinfold (vanessapinfold@mcpin.org)

Research funding applications submitted to the National Institute of Health Research (NIHR) in England require teams to evidence the extent to which patients and the public have been involved in developing a proposal, and whether they will be involved once a grant is awarded. Three levels of involvement have been commonly identified: consultation; collaboration; and, control. Patient and Public Involvement (PPI) initiatives are often assessed against these benchmarks.

PARTNERS2 is a NIHR five year multi-site study with collaborators bringing different expertise to the team: trial management, statistical, clinical, qualitative methods, outcome measurement and expertise from experience. The aim of the research is to deliver and test collaborative care for people with ongoing mental health needs in primary care settings.

Originally, the study was designed with a PPI programme embedded across the study including a PPI Lead and coordinator, 3 local lived experience advisory panels (LEAP) made up of mental health service users and carers, plus 3 service user researchers. Expertise from experience was a specific asset held by certain team members, and reflected in job titles.

Three years on, the team have learned about what works well, and also the difficulties of their planned PPI approach. The original starting point of structured collaboration has developed the study focus into one of a co-production model. Expertise from experience is further dispersed across the study, and roles have changed leaving little distinction between a project researcher and a service user researcher. The team are continually attempting to adapt and evaluate their involvement practices to increase their relevance, benefit and effectiveness for the programme as a whole. Co-produced decision making has led to the selection of outcomes measures and the development of a bespoke trial website with team and LEAP members featuring in videos making the case for research and participation in the trial. A way of working document was produced and has been updated, setting out principles for co-production in PARTNERS2. As new members join, reflecting on these agreed principles is particularly important. A research participant charter is under construction to provide the research team with jointly agreed standards for how they aim to support research participants to ensure people have a positive experience of the PARTNERS2 research study.

Understanding how to best integrate expertise from experience within mental health research teams is fundamental for developing coproduced mental health research. Sharing power across a diverse multi-site research programme is challenging. It is also possible.

Trial registration number: we are awaiting registration.

**Acknowledgements**

The entire PARTNERS2 research team working across universities of Birmingham, Lancaster, Plymouth, Exeter, Manchester, and Warwick.

## O19 Developing resources for a learner-centred approach to learning and development for public involvement in research

### Lucy Frith^1^, Bernard Gudgin^2^, Amander Wellings^3^

#### ^1^National Institute for Health Research (NIHR), Research Design Service North West, University of Liverpool, Liverpool, UK; ^2^Public representative, Thames Valley, UK; ^3^Public representative, Norfolk, UK

##### **Correspondence:** Lucy Frith (L.J.Frith@liverpool.ac.uk)

In 2015 the National Institute for Health Research (NIHR) ‘Going the Extra Mile’ report highlighted the need to improve support for learning and development, which was reiterated in an NIHR-wide Learning & Development Working Group report of 2015 [1]. Despite widespread training activity, resources that support members of the public, researchers and public involvement managers can be difficult to find, and opportunities often are insufficiently promoted or duplicated.

The INVOLVE Learning and Development Project Group was formed in 2016 to improve the resources and support available for learning and development. Consisting of public members, and public involvement leads from the charity sector and a variety of NIHR organisations, the Group took a collaborative approach and formed six sub-groups to address: individuals’ learning needs; inductions; how opportunities are promoted and accessed; how to share learning about engaging diverse communities; how resources are shared across websites; and top tips to promote good practice.

Developing resources in these areas will allow individuals to assess their own skills and knowledge, how this relates to the needs of their role, and what areas require development. Improved information at inductions will benefit those new to research or involvement, and better accessibility of learning opportunities will prevent existing duplication.

This presentation will include an interactive session during which delegates will use some of the new learner-centred resources. By the end of the session, delegates will have an understanding of the resources available, where they can be found and how they might be used to support their own (and others’) development.

**Acknowledgements**

This presentation will be delivered on behalf of the INVOLVE Learning and Development Project Group.

**References**

1. INVOLVE (2015) NIHR-wide learning and development for public involvement: working group report and recommendations, INVOLVE: Eastleigh. Publication link: http://www.invo.org.uk/wp-content/uploads/2016/03/FINAL-NIHR-LD-report-July-2015.pdf

## O20 The East Midlands Sharebank – a cross-institutional model for sustainable learning and development for public involvement in research

### Adele Horobin^1,2^, Colleen Ewart^3^, Fred Higton^3^, Stevie Vanhegan^3^, Raksha Pandya-Wood^4^, Jane Stewart^5^, Andy Wragg^2,6^, Paula Wray^7^, Kirsty Widdowson^2^

#### ^1^National Institute for Health Research (NIHR) Nottingham Biomedical Research Centre, Nottingham, UK; ^2^Nottingham University Hospitals NHS Trust, Queens Medical Centre, Nottingham, UK; ^3^Public representative, Nottingham, UK; ^4^National Institute for Health Research (NIHR) East Midlands Research Design Service, Department of Health Sciences, University of Leicester, Leicester, UK; ^5^National Institute for Health Research (NIHR) East Midlands Research Design Service, School of Medicine, University of Nottingham, Nottingham Health Science Partners, Queen's Medical Centre, Nottingham, UK; ^6^National Institute for Health Research (NIHR) Nottingham Biomedical Research Centre, Queen’s Medical Centre, Nottingham, UK; ^7^INVOLVE Coordinating Centre, University of Southampton, Southampton, UK

##### **Correspondence:** Adele Horobin (adele.horobin@nottingham.ac.uk)

**Aims of the session**

- To describe the Sharebank model, why and how it was developed.
- To discuss how public involvement training can be co-produced with the public.
- To provide an opportunity for the audience to give their views on the Sharebank model and whether this is something they would consider for their own region.

**Why is it important and to whom?**

The National Institute for Health Research (NIHR) ‘Going the Extra Mile’ report recommends that the public and researchers should be better supported to do public involvement and that local organisations should work together to do this. We have developed a cross-institutional model, from the ‘grass roots’, to fulfil these recommendations. It is of strategic importance to NIHR and of interest to public involvement leads, public and researchers.

**What difference has, or could, this project make?**

The Sharebank has brought NIHR and NHS organisations together, aligning strategic objectives for public involvement support and helping public and researchers to share their experiences. It provides the means for organisations to share training and resources for public involvement at minimal cost.

This session will inspire the audience in:

- Collaborating to create flexible, sustainable learning and development programmes for public involvement in research in their regions.
- How to involve members of the public in co-producing public involvement training.

**What will people take away from your session?**

- Guidance: describing a pathway for other organisations to work closely together in learning and development for public involvement.
- Contacts: we would be happy to advise anyone in setting up their own ‘Sharebank’.

## O21 Taking involvement online: development and evaluation of an online forum for patient and public involvement in palliative care research

### Lisa Jane Brighton^1^, Sophie Pask^1^, Hamid Benalia^1^, Sylvia Bailey^1^, Marion Sumerfield^1^, Simon Etkind^1^, Fliss E. M. Murtagh^1,2^, Jonathan Koffman^1^, Catherine J. Evans^1,3^

#### ^1^Department of Palliative Care, Policy and Rehabilitation, Cicely Saunders Institute, King’s College London, London, UK; ^2^Wolfson Palliative Care Research Centre, Hull York Medical School, University of Hull, Hull, UK; ^3^Department of Palliative Medicine, Sussex Community NHS Foundation Trust, Brighton, UK

##### **Correspondence:** Lisa J. Brighton (Lisa.brighton@kcl.ac.uk)

**Aim of the presentation**

To share learning from the development and evaluation of an online forum for patient and public involvement (PPI) in palliative care research: CSI Public Involvement [www.csipublicinvolvement.co.uk].

**Why is it important and to whom?**

INVOLVE highlights the importance of ensuring PPI approaches are accessible, fair, responsive, and supportive. This is particularly important in populations where, due to advanced illness or caring responsibilities, face-to-face involvement becomes challenging. In response to this problem and to contribute towards an evidence base around virtual PPI, we have developed and evaluated an online forum for PPI in palliative care research, in collaboration with existing PPI members via a consultation process.

**What difference has, or could, this project make?**

Developing and evaluating the online forum has led to three key differences: increased coproduction skills, improved research quality, and a new knowledge base for developing online PPI platforms. This experience encouraged a shift from collaboration to coproduction, to ensure the success of the forum. Input from new PPI members has improved our research quality through more diverse involvement and feedback, e.g. different people’s experiences in relation to ‘difficult conversations’ and ‘feeling safe’. Evaluation of this forum using focus groups and online questionnaires has resulted in new knowledge of how best to engage and sustain online forums in PPI, and empower PPI members and researchers in using this co- productive method.

**What will people take away from your presentation?**

Based on our research, attendees will receive guidance on both developing and evaluating an online forum for PPI in research.

**Acknowledgements**

This study was funded by the King’s College London and Wellcome Trust Institutional Strategic Support Fund, and the National Institute for Health Research (NIHR) Collaboration for Leadership and Applied Health Research and Care (CLAHRC) South London. The views expressed in this publication are those of the authors and not necessarily those of the NHS, the NIHR, or the Department of Health.

## O23 Partnering to improve BME access, inclusion and involvement in research: exchange visits with third-sector organisations for shared learning and improvement

### Susan Hrisos^1^, Julie Marshall^2^, Lyndsay Yarde^3^, Bren Riley^4^, Paul Whitlock^5^, Jacqui Jobson^5^, Safia Ahmed^6^, Judith Rankin^1^, Lydia Michie^1^, Jason Scott^1^

#### ^1^Institute of Health & Society, Newcastle University, Newcastle upon Tyne, UK; ^2^InvolveNorthEast, Newcastle upon Tyne, UK; ^3^Healthwatch Newcastle, Newcastle upon Tyne, UK; ^4^Riverside Project, Newcastle upon Tyne, UK; ^5^Advocacy Centre North, Newcastle upon Tyne, UK; ^6^Health and Race Equality Forum, Newcastle upon Tyne, UK

##### **Correspondence:** Susan Hrisos (susan.hrisos@ncl.ac.uk)

**Background**

People from black minority and ethnic (BME) groups are under-represented in research aiming to improve health services, healthcare provision and patient safety, limiting the relevance and generalisability of research based on ‘mainstream’ populations. This may widen existing inequalities in health, access to health care, and hinder meaningful involvement in healthcare and treatment decisions. Community groups and organisations are often more successful in engaging and consulting with people who researchers can consider ‘hard to reach’, but their methods can lack the rigour that is central to formal research.

**Methods**

This collaboration of university-based researchers and local community partners identified a mutual benefit from shared learning, so we ran an exchange visit programme during which we spent a period of time within each other’s organisations to observe what we each do, including how we go about priority setting and how we gather, evaluate and report data. The aim was for everyone involved to reflect on and improve what we do respectively, and to find a common-ground methodology for improving access, inclusion and evaluation of more meaningful research.

**Findings**

This presentation will describe the shared learning and participatory approach used during two successive projects during which we a) co-designed and piloted an approach to enabling BME participation in research, b) created a blue-print for a sustainable model of community-based support for inclusive research, and c) a learning and development toolkit to promote capacity to evaluate within community partner organisations and to promote engagement and consultation skills of researchers in relation to minority populations.

## P1 Working together to better represent the unheard voices of 16-24 year olds in health research

### Caroline R. Barker^1^, Megan Barlow-Pay^2^, Aisha Kekere-Ekun^3^, Aniqa Mazumder^3^, Aniqa Nishat^3^, Rebecca Petley^3^

#### ^1^National Institute for Health Research Southampton Clinical Research Facility and Biomedical Research Centre, University Hospital Southampton NHS Foundation Trust, Southampton, Hampshire, UK; ^2^National Institute for Health Research Design Service South Central, University of Southampton, Southampton, Hampshire, UK; ^3^Young Adult Patient and Public Involvement Group member, Southampton, Hampshire, UK

##### **Correspondence:** Caroline R. Barker (YAPPI@nihr.ac.uk)

This poster shares experiences of recruitment, facilitation and sustainability of patient and public involvement (PPI) with an underrepresented audience – 16-24 year olds. Co- produced by young adults, it reflects on the challenges and potential solutions of involving this hard to reach group in research.

The Young Adult PPI (YAPPI) Group is a joint initiative between the Southampton NIHR Clinical Research Facility, NIHR Biomedical Research Centre and NIHR Research Design Service South Central. This recently established group was formed to address the unmet needs of this particular demographic. This group is open to researcher contact and has contributed to several pieces of pre-funding study development work.

Young people with chronic conditions go through a transitional care period and move from child-centred to adult-centred health services. While transitional care is being addressed in the clinical setting, there is little to address the difficulties associated with the transition in health and social care research. Additionally, this age range faces novel issues such as higher education, leaving home, starting careers or relationships. These challenges influence their clinical and social needs as well as their personal priorities; their involvement is therefore invaluable in delivering appropriate health research.

Our young adult representatives will share their advice on how to engage with and promote PPI activities for this population. Drawing on their experiences they will cover: how to approach this demographic; what attracts this age range to be involved and how to facilitate and maintain communication with representatives.

## P2 “We’ve walked the walk”. Lessons from involving young people with experience of substance misuse services in a research study

### Louca-Mai Brady^1^, Lorna Templeton^2^

#### ^1^Kingston and St George’s Joint Faculty and Independent Research Consultant. London, UK; ^2^Independent Research Consultant. Bristol, UK

##### **Correspondence:** Louca-Mai Brady (loucamai.brady@gmail.com)

The Youth Social Behaviour and Network Therapy (Y-SBNT) study was a National Institute for Health Research (NIHR) funded randomised controlled trial. The study aimed to demonstrate the feasibility of recruiting young people to a family- and wider social network- based intervention [1] by testing an adapted version of an established adult intervention (SBNT) [2]. The study was also a case study in doctoral research by one presenter (L-MB) on how young people’s involvement can be embedded in health services and research [3] This poster draws on this doctoral research and the study report [4] to outline how a group of young advisors who had used drug and alcohol services in the past worked with the research team to make sure that the research was relevant to young people. The young advisors were involved in the design of key research documents and tools, data analysis and interpretation and reporting. But there were some challenges in recruiting and working with this group of young people, and we found that the standard ‘young people’s advisory group’ model did not work for many of the young people we were trying to engage. This has informed wider learning on how best to involve a group of young people who are ‘less frequently heard’, and led to the development of a different model of public involvement. The poster outlines the model which emerged from this study, which explores whether traditional models of public involvement can potentially exclude some of the young people most likely to use health services, and identified the potential for new flexible and young people-centred approaches to involvement in research.

It will be of interest to those involving children and young people in research as well as those with an interest in methods for including more diverse voices in public involvement beyond the ‘usual suspects’.

**Acknowledgements**

Our grateful thanks to all the young people who have been involved in the project, all the services and staff who supported their involvement, and to the members of study team not involved in the development of this poster including the Principal Investigator Alex Copello and Sangeeta Ambegaokar, Donna Back, Ed Day, Charlie Lloyd, Eilish Gilvarry, Paul McArdle and Judith Watson.

The Y-SBNT project was funded by the National Institute for Health Research (NIHR) Health Technology Assessment Programme (project number 11/60/01). The views and opinions expressed herein are those of the authors and do not necessarily reflect those of the Health Technology Assessment Programme, NIHR, NHS or the Department of Health. The study was coordinated by the University of Birmingham and sponsored by Birmingham and Solihull Mental Health Foundation Trust.

**References**

1. Watson. J., Back, D., Toner, P., Lloyd, C., Day, E., Brady, L.M., Templeton, L., Ambegaokar, S., Parrott, S., Torgerson. D., Cocks, K., Gilvarry, E., McArdle, P., Copello, A. (2015), A randomised controlled feasibility trial of family and social network intervention for young people who misuse alcohol and drugs: study protocol (Y-SBNT). Pilot and Feasibility Studies, Vol. 1 No. 8, available at: https://pilotfeasibilitystudies.biomedcentral.com/articles/10.1186/s40814-015-0004-4

2. Copello, A., Orford, J., Hodgson, R. and Tober, G. (2009), Social Behaviour and Network Therapy for Alcohol Problems, Routledge, London.

3. Brady, L.-M. (2017), Rhetoric to reality: An inquiry into embedding young people's participation in health services and research. PhD, University of the West of England, available at: http://eprints.uwe.ac.uk/29885

4. Watson, J., Toner, P., Day, E,. Back, D., Brady, L.M., Fairhurst, C., Renwick, C., Templeton, L., Akhtar, S., Lloyd, C., Li, J., Cocks, K., Ambegaokar, S., Parrott, S., McArdle, P., Gilvarry, E. and Copello, A. (2017), Youth social behaviour and network therapy (Y-SBNT): adaptation of a family and social network intervention for young people who misuse alcohol and drugs a randomised controlled feasibility trial. Health Technology Assessment, Vol. 21 No. 15, available at: https://www.journalslibrary.nihr.ac.uk/hta/hta21150/#/abstract

## P3 Children and young people involvement in an evidence synthesis on mental health in children and young people with long-term conditions

### Erin Walker^1^, Darren Moore^2^, Liz Shaw^2^, Michael Nunns^2^, Jo Thompson Coon^2^

#### ^1^Centre for Outcomes and Experiences Research in Child Health, Illness and Disease, Great Ormond Street Hospital for Children NHS Foundation Trust, London, UK; ^2^National Institute of Health Research Peninsula Collaboration for Leadership in Applied Health Research & Care, University of Exeter Medical School, Exeter, UK

##### **Correspondence:** Erin Walker (erin.walker@gosh.nhs.uk)

**Aims of the session**

Whilst encouraged by research funders, it is unusual to involve patients and the public in systematic reviews. We involved children and young people (CYP) with relevant experience, in a large National Institute of Health Research funded evidence synthesis project of mental health interventions for children and young people with long–term conditions. The aim of this session is to share our experiences of the project from the perspectives of the CYP and the research team.

**Why is it important and to whom?**

The involvement of CYP in this project has had a powerful and meaningful impact not only on the research but also on the review team and the CYP themselves. By sharing our experiences, we hope to demonstrate that with careful planning and appropriate resources, involving CYP in systematic reviews is both feasible and practical.

**What difference has the project made?**

Overall, the involvement of CYP provided a grounding for the research in real-world experience. Specifically, CYP were involved in validating research ideas, confirming appropriate search terms, producing suggestions for search sources, interpreting review findings from a different vantage point, and producing a plain language summary and a podcast for dissemination of key messages.

**What will people take away from the session?**

Involving CYP and parents in systematic review projects is novel but achievable and rewarding. Involvement should form part of core systematic review methods. The voices of CYP are often neglected in research but can be invaluable in making sense of large amounts of complex information.

**Acknowledgments**

Our group of children and young people who contributed include Katie Hughes, Katrina Brooks, Summer Teale, Harry Lockhart, Charlotte Rushton, Miriam Meziane, Thomas Gardner, Benjamin Meziane

## P7 Solutions for PPI challenges in studies of acute disease, stigmatised behaviours and disenfranchised populations: Lessons from sexual health research

### Paula Blomquist^1,2^, Sarah Cochrane^3^, Natalie Edelman^4,5^, Josina Calliste^2^, Jackie Cassell^2,4^

#### ^1^Public Health England, London, UK; ^2^National Institute for Health Research (NIHR) Health Protection Research Unit in Blood Borne and Sexually Transmitted Infections at UCL, London, UK; ^3^University Hospitals Bristol NHS Foundation Trust, Bristol, UK; ^4^Brighton & Sussex Medical School, Brighton, UK; ^5^University of Brighton, Brighton, UK

##### **Correspondence:** Paula Blomquist (Paula.Blomquist@phe.gov.uk)

Patient and Public Involvement (PPI) is an increasingly routine aspect of health research, however challenges remain. Much PPI guidance focuses on studies of chronic diseases, where access to members of the public for PPI is enabled by patient support groups or because patients are continuously engaged with health care.

In contrast, there is little PPI guidance for studies of acute disease, socially disenfranchised groups, or populations defined by stigmatised behaviours or diagnoses. In these scenarios members of the public may be less accessible to researchers and less interested in PPI. Much sexual health research fits into one or more of these criteria.

The aim of this presentation is to address the gap in PPI guidance by considering possible solutions to challenging PPI scenarios. We present lessons learned from four studies affiliated to the NIHR’s Health Protection Research Unit in Blood Borne and Sexually Transmitted Infections, which focus on sexual health, ethnic minorities, and young people. Our tips focus on three stages of PPI: defining a target PPI group, accessing patients or members of the public, and engaging with those who have agreed to take part in PPI. We take a practical approach, with advice ranging from how to create more appealing patient invitations at health providers, to ways to engage with patients with particular concerns about confidentiality and anonymity.

Sharing these experiences may help researchers in sexual health and other fields have a greater understanding of potential challenges in PPI and how to overcome them.

## P9 Inverting the Patient Involvement Paradigm: Establishment of the Patient Led Research Hub

### Laura B. Mader^1,2^, Sabine Kläger^2^, Ian B. Wilkinson^1^, Thomas F. Hiemstra^1,2^

#### ^1^School of Clinical Medicine, University of Cambridge, Cambridge, UK; ^2^Cambridge Clinical Trials Unit, Cambridge University Hospitals NHS Foundation Trust, Cambridge, UK

##### **Correspondence:** Thomas F. Hiemstra (tfh24@cam.ac.uk)

The NIHR has pushed Patient and Public Involvement in healthcare to the forefront of research priorities. Many initiatives have proven successful, and interested members of the public are now able to participate in a breadth of opportunities. However, involvement is generally limited to consultation on study design or promoting participant adherence.

Further, a clearly documented mismatch between patient demand and actual researched interventions exists, with most funded studies prioritising drug trials [1]. Quality of life and fundamental priorities as described by patients and carers are often ignored.

A pioneering initiative launched in May 2015 by the Cambridge Clinical Trials Unit assumes this role. The Patient Led Research Hub (PLRH) supports research projects emerging directly from, and proposed by, patient organisations: the only UK initiative to allow patients to develop and conduct research in this manner. Any research proposal, irrespective of its focus or disease area, is considered and feasible projects are supported with resources and expertise in design and delivery.

Proposers maintain co-ownership of the project and intellectual property where relevant, ensuring research remains relevant and credible. Equal collaboration underpins competitive grant applications to public funders, research charities or industry partners. If funding is obtained, projects become autonomous to the extent possible, allowing PLRH resources to become available for new proposals.

Thus far, the PLRH has received excellent feedback, with 23 proposals from 19 different organisations and independent sources. Proposals are wide-ranging from bench to bedside, but with a primary focus on quality of life. Three projects are active, one funding bid is in preparation, three proposals have been linked with ongoing, aligned research work, and nine proposals are in various stages of work-up. Further investment is now required to increase capacity and infrastructure to support incoming proposals.

**References**

1. Crowe S, Fenton M, Hall M, *et al.* Patients’, clinicians’ and the research communities’ priorities for treatment research: there is an important mismatch. Research Involvement and Engagement 2015;**1**:2. doi:10.1186/s40900-015-0003-x

## P10 Building on a partnership’s experiences from coordinating involvement in health and social care education to developing user-led research: lessons learnt

### Mel Hughes^1^, Angela Warren^2^, Peter Atkins^2^, Hazel Eaton^3^

#### ^1^Faculty of Health and Social Sciences, Bournemouth University, Bournemouth, UK; ^2^PIER (Public Involvement in Education and Research) partnership, Bournemouth University, Bournemouth, UK; ^3^Research and Development, Dorset Healthcare University NHS Foundation Trust, Dorset, UK

##### **Correspondence:** Mel Hughes (mhughes@bournemouth.ac.uk)

The aim of the session is to share our critical reflections and insights from developing user- led research and seeking to establish a positive and collaborative culture and environment for involving experts by experience in health and social care research.

The PIER partnership is an established university service user and carer partnership with over 90 active members, 900 hours of direct involvement activity a year, and a history of collaborating with community organisations to enhance health and social work education. Since 2015 we have sought to draw on this expertise to develop a culture within the university and with our external research partners for engaging meaningfully in PPI and in particular, user-led research. From our network of involvement coordinators across the UK, we know that many organisations (universities, charitable organisations, health and social care providers) are seeking to develop user-involved and led research and to identify strategies and opportunities for doing so meaningfully and with the greatest impact. We consider ourselves at an early stage of developing this role and are keen to contribute to national developments by sharing our activities, efforts, mistakes and learning to date, and to seek guidance from others.

The session will benefit anyone seeking to explore and reflect on the processes involved. We will summarise: our exploration and evaluation of different models of PPI and our emerging evidenced based framework for user-led research; key points and stages of learning; and our top tips for organisations embarking on a similar journey.

## P13 Embedding patient and public involvement (PPI) in a regional research network and beyond: findings and action points from the IMPRESS project and Collaboration for Leadership in Applied Health Research and Care (CLAHRC) East of England

### Julia Keenan^1^, Fiona Poland^1^, Helena Wythe^2^, Amander Wellings^3^, Penny Vicary^3^

#### ^1^University of East Anglia, Norwich, Norfolk, UK; ^2^University of Hertfordshire, Hatfield, Hertfordshire, UK; ^3^Members of the Patient and Public in Research Group (PPIRes), NHS South Norfolk Clinical Commissioning Group, Norwich, Norfolk, UK

##### **Correspondence:** Fiona Poland (f.poland@herts.ac.uk)

**Background**

We share findings from an action research project (IMPRESS: Implementing PPI in an NHS Research Programme: Evaluating the PPI contribution to CLAHRC research implementation) which studied how PPI has been implemented within a regional, applied research programme (a CLAHRC: Collaboration for Leadership in Applied Health Research and Care). This builds on findings from a previous national study (RAPPORT: ReseArch with Patient and Public invOlvement: a RealisT evaluation). Our project team includes two PPI co-researchers and an advisory group with a lay chair and further PPI representatives. IMPRESS employed a theoretical framework to explore in-depth, the experiences of PPI within the CLAHRC programme, from different points of view. Our findings identified the barriers and facilitators to the programme’s aim of ‘fully embedded, active and comprehensive’ PPI which then inform ten key action points for developing PPI in a programme.

The network of CLAHRCs are planned to play a key role in co-developing and co-delivering NIHR’s PPI strategy across regions in England. The CLAHRC studied here makes policy and resource commitments to PPI, has PPI as a research theme and works in partnership with regional PPI networks. It is thus important to report systematically researched findings on processes and outcomes of this commitment, both to inform specific local action and to report broader conceptual lessons for PPI knowledge and practice.

We detail, with illustrative examples, how 10 case study projects made sense of PPI, bought into PPI, enacted PPI and appraised PPI. The action research approach enables, actions and solutions to problems of embedding PPI to be ‘fine-tuned’ in further research cycles to evidence and enact sustainable PPI processes and outcomes for all stakeholders.

See a film of the study results at: https://www.youtube.com/watch?v=sL9EbvYmaxA

**Acknowledgements**

Wider IMPRESS team members: Amanda Howe, Jonathan Boote, Anna Varley, study advisory group members

## P14 Creating a regional network for public involvement in research linked to regional, national and international good practice

### Carol Rhodes^1^, Magdalena Skrybrant^2^, Steven Blackburn^1^, Lucy Chatwin^3^, Mary-Anne Darby^4^, Andrew Entwistle^4^, Diana Hull^5^, Naimh Quann^5^, Gary Hickey^6^, Krysia Dziedzic^1^

#### ^1^NIHR Research Design Service West Midlands, Research Institute for Primary Care & Health Sciences, Keele University, Staffordshire, UK; ^2^NIHR Collaboration for Leadership in Health Research and Care West Midlands, Institute of Applied Health Research, University of Birmingham, Birmingham, UK; ^3^Academic Health Science Network West Midlands, Institute of Translational Medicine, Queen Elizabeth Hospital, Birmingham, UK; ^4^NIHR Clinical Research Network West Midlands, Greyfriars Business Park, Stafford, UK; ^5^NIHR/Wellcome Trust Birmingham Clinical Research Facility, University Hospitals Birmingham NHS Foundation Trust, Birmingham, UK; ^6^INVOLVE, University of Southampton Science Park, Southampton, UK

##### **Correspondence:** Carol Rhodes (c.a.rhodes@keele.ac.uk)

**Background**

The National Institute of Health Research (NIHR), through the INVOLVE organisation, aims to galvanise regional networks supporting public involvement in research and facilitate closer working to enable sharing of expertise, good practice and innovation. [1, 2]

We aimed to create and sustain a regional network for public involvement in the West Midlands.

**Methods**

We will describe:

- The creation of the regional network: PILAR (Public Involvement and Lay Accountability in Research and Innovation)
- The planning and hosting of our first conference, ‘Health Research: Better Together’, bringing together organisations in the region involving the public in research and innovation.
- Using an interactive workshop to identify future regional priorities for public involvement.

**Results**

PILAR, the regional network for the West Midlands, was established in 2013. It comprises lay and professional members from NIHR and other partner organisations set up to pool expertise and share good practice, PILAR provides leadership for public involvement in the region. Following our inaugural conference, we have established our ‘PILAR Pledge’. These were based on the co-developed priorities for public involvement in the region identified in an interactive workshop during the PILAR conference. Subsequently, PILAR has created the foundation for a collaborative community across the West Midlands whose activities are closely linked to INVOLVE and aligned strategically with the NIHR’s ‘Going The Extra Mile’ recommendations.[2] For example, PILAR has begun to identify and share learning and development opportunities for public involvement for professional and lay people in the region.

**Conclusion**

Regional networks can play a pivotal role in ensuring meaningful public involvement can be achieved. A strong action plan developed following our event will ensure we can tackle common and challenging issues together. Working together at a regional level can help shape better public involvement in health research, service and innovation.

**Acknowledgement**

This work is presented on behalf of the Public Involvement and Lay Accountability in Research and Innovation regional network for the West Midlands. The authors would like to thank Gary Hickey and NIHR INVOLVE for their support for PILAR. Time for Krysia Dziedic is part funded by a NIHR Knowledge Mobilisation Research Fellowship (KMRF-2014-03- 002). Magda Skrybrant is part funded by Collaborations for Leadership in Applied Research and Care West Midlands. Steven Blackburn is funded by the Research Design Service.

**References**

1. INVOLVE. Examples of regional networks for public involvement in research. 2016. http://www.invo.org.uk/wp-content/uploads/2016/03/INVOLVE-Regional-Networks-report- 2016.pdf. Accessed 12th September 2017

2. National Institute for Health Research. Going the extra mile: Improving the nation’s health and wellbeing through public involvement in research. 2015. https://www.nihr.ac.uk/news/going-the-extra-mile-a-strategic-review-of-public-involvement-in- the-national-institute-for-health-research/2739. Accessed 12th September 2017

## P15 Co-production in practice: Service users co-develop instruments to measure and evaluate the impact of their involvement

### Sabrina A. Eltringham, Jim Gordon, Sue Franklin

#### Directorate of Therapeutics and Palliative, Sheffield Teaching Hospitals NHS Foundation Trust, Sheffield, UK

**Background**

Accounts of impact can be improved by involving service users [1]. This poster will describe how service users of a generic rehabilitation research panel co-produced tools to measure and evaluate impact. This is important because it respects the equality and different roles and perspectives of those involved in developing measures of accountability [2,3].

**Method**

We reviewed evidenced based frameworks and guidelines [4,5], and with the support of our research organisation and other panels, co-developed qualitative questionnaires to identify values associated with patient and public involvement. Our members piloted service user and researcher questionnaires and shared our instrument to collect quantitative metrics. Amendments to the questionnaires were collectively agreed. We continue to implement initiatives relevant to our local context and share these with our colleagues.

**What difference has this project made?**

- Service user involvement has ensured that measurement is meaningful and outcomes which are important to service users are included.
- The instruments have improved our understanding of what aspects of involvement work for whom, and in what circumstances.
- Provided pragmatic evidence to support continued funding of our Patient Panel at a time of financial constraints.

**Key learning points**

- How to work collaboratively to maintain relevance to the local context of service user involvement whilst achieving organisational objectives.
- Implementing guidance on measuring impact, which captures the perspectives of different stakeholders.
- Measuring, comparing and understanding impact for different stages of the research process and how it makes a difference for different groups.

**References**

1. Staniszewska et al. Developing the evidence base of patient and public involvement in health and social care research: the case for measuring impact. International Journal of Consumer Studies. 2011; 35:628-632.

2. INVOLVE. Public involvement in research: values and principles framework. INVOVE: Eastleigh;2015.

3. INVOLVE. Co-production: Old wine in new bottles or vintage PPI? Available from: http://www.invo.org.uk/wp- content/uploads/2017/02/INVOLVENewsletterwinter2016-17FINAL-1.pdf [Accessed 11th September 2017].

4. Popay, J and Collins, M (editors) with the PiiAF Study Group. The Public Involvement Impact Assessment Framework Guidance. Universities of Lancaster, Liverpool and Exeter; 2014.

5. Staley, K. Summary Exploring Impact: Public Involvement in NHS, public health and social care research. INVOLVE, Eastleigh; 2009.

## P16 Preventing post-operative urinary retention: an example of researcher-patient co-production

### Joni Jackson^1,2^, Nick Leggett^1,2^, Philippa Davies^1,2^, Manjula Nugawela^1,2^, Lauren Scott^1,2^, Verity Leach^1,2^, Alison Richards^1,2^, Anthony Blacker^3^, Paul Abrams^4^, Jitin Sharma^3^, Jenny Donovan^1,2^, Penny Whiting^1,2^

#### ^1^The National Institute for Health Research Collaboration for Leadership in Applied Health Research and Care West (NIHR CLAHRC West) at University Hospitals Bristol NHS Foundation Trust, Bristol, UK; ^2^Population Health Sciences, Bristol Medical School, University of Bristol, Bristol, UK; ^3^University Hospitals Coventry and Warwickshire, Coventry, UK; ^4^North Bristol Trust, Bristol, UK

##### **Correspondence:** Penny Whiting (penny.whiting@bristol.ac.uk)

Interest in patient and public involvement (PPI) in research is increasing, yet evidence about its impact on health research is limited. We present our experience of successful public involvement on a project developed from a patient submitted research idea, describing the role of our public contributor in the initiation, design, production, distribution and evaluation of a project aimed at preventing post-operative urinary retention (PO-UR).

The research idea was submitted through the CLAHRC West open call in 2014 by Nick Leggett (NL), who had previously experienced PO-UR as a surgical patient. Taking on the role of co-Principal Investigator, NL attended meetings of the research team as well as co-chairing wider advisory group meetings. Offered training in research methodology, NL was included at all stages of the project, acting as systematic reviewer through to the design of the primary study.

Being a common outcome of frequently performed operations (incidence of 10.7% - 84% after joint replacement), PO-UR affects many patients. Untreated, PO-UR can lead to complications such as infection due to urinary stasis and acute kidney injury, resulting in delayed hospital discharge and a need for additional post-hospitalisation care. This project highlighted a difference in attitudes between patients and clinicians, in relation to the treatment of PO-UR. Clinicians sometimes consider PO-UR to be a minor problem, easily solvable by catheterisation. From the patient’s perspective however, this invasive procedure is often considered an undesirable solution, with risks of catheter-associated complications (e.g. urinary tract infection) and distress for the patient. This project provides an example of potentially impactful research, unlikely to have been developed if not for patient involvement in co-initiation.

NL’s continued involvement throughout has added unique knowledge, personal insight and relevance to the project. Not only encouraging for public involvement in future research, this project provides insight into the benefits and challenges that can be encountered by both researchers and public contributors during the cycle of coproduction.

PROSPERO ID:

CRD42016051030; CRD42016048765

Project link: https://clahrc-west.nihr.ac.uk/research/projects/preventing-post-operative-urinary-retention-improve-outcomes-reduce-costs/

**Acknowledgements**

The research is supported by the National Institute for Health Research (NIHR) Collaboration for Leadership in Applied Health Research and Care West (CLAHRC West) at University Hospitals Bristol

## P18 The critical role of patients, parents and carers in guiding the paediatric rheumatology research strategy for the United Kingdom through the clinical studies group

### Simon R. Stones, Catherine Wright

#### NIHR: CRN Children/Arthritis Research UK Paediatric Rheumatology Clinical Studies Group, Liverpool, UK

##### **Correspondence:** Simon R. Stones (simonstones@icloud.com)

**Background**

The insights and experiences of young people with long-term health conditions, such as musculoskeletal diseases, in addition to their parents and carers, must inform and shape all aspects of health research. Lived experiences are critical for successful research in many ways. In turn, it is possible that the involvement of young people, parents and carers may enhance expectations and subsequent levels of satisfaction when individuals participate in research. The primary aim of young people, parent and carer representatives on the United Kingdom’s paediatric rheumatology clinical studies group is to provide strategic guidance on how to effectively incorporate the views of young people, parents and carers in rheumatology clinical and health services research.

**Materials and methods**

Young people, parents and carers actively contribute to the clinical studies group in a number of ways, and lead on a series of internal and external initiatives. In order for representatives to capture the wider views of young people with musculoskeletal diseases, as well as their parents and carers, representatives must exemplify the current views within the wider community. The representatives do this through networking, research prioritisation exercises, liaison with external stakeholder groups, and through project research meetings.

**Results**

Young people, parent and carer representatives actively contribute to monthly meetings and are often appointed to advise external project meetings. Representatives are also members of various study steering committees, ensuring that the voice of young people, parents and carers is embedded into the culture of research activities, as well as bridging the gap between the research community, patient groups and charities. Furthermore, young people, parents and carers have been involved in formulating their own research priorities as part of the wider paediatric rheumatology research strategy [1].

**Conclusions**

By widely using the voice of young people, parents and carers as a catalyst for high quality, young person- and family-focused research, it is hoped that the often-negative experiences of living with long-term conditions such as musculoskeletal diseases, can be used to positively shape research, and therefore, contribute to the best possible care, treatment and support for young people and their families living with musculoskeletal disease in the near and distant future.

**Acknowledgments**

This abstract is presented on behalf of the lay/consumer representatives of the paediatric rheumatology clinical studies group. The authors would like to acknowledge all members of the clinical studies group, past and present, for their continued support for young people, parents and carers.

**References**

1. Beresford M. NIHR CRN: Children/Arthritis Research UK Paediatric Rheumatology Clinical Studies Group (CSG) Clinical Research Strategy. Arthritis Research UK. 2015.

## P20 What can we learn from a learning exchange? The value, approach and ongoing benefits

### Kate Boddy^1^, Jenny Irvine^2^, Jim Harris^3^, Neil Joseph^4^

#### ^1^NIHR CLAHRC South West Peninsula (PenCLAHRC), University of Exeter Medical School, Exeter, UK; ^2^NIHR CLAHRC North West Coast (CLAHRC NWC), Based at Division of Health Research, Lancaster University, Lancaster, UK; ^3^Peninsula Public Involvement Group (PenPIG), PenCLAHRC, South West Peninsula, Exeter, UK; ^4^Public Reference Panel (PRP), CLAHRC NWC, North West Coast area, Liverpool, UK

##### **Correspondence:** Kate Boddy (K.boddy@exeter.ac.uk)

There are established principles for meaningful involvement, but less understanding of how these principles can be carried out in practice and actualised into involvement plans and actions for health research. As part of a national collaboration, operating in distinct regions across the UK, we offer a comparison between the two organisations’ different approaches to an overarching goal and the outcomes from these.

Our aim is to illustrate how two organisations, with distinct models for public involvement in health research, developed and improved their involvement practices through a learning exchange. Public advisors provide insights about the experience of taking part in the exchange and the impact of the learning on practices

The process of the learning exchange, which included over 30 public advisors at some stages, will also be explored and will be of importance to anyone with an interest in learning and development in involvement.

The two organisations were able to provide practical examples of ‘how to do’ public involvement, bringing involvement principles to life. Through this learning process the two groups were able to reflect on their own practices enabling new ideas and procedures to be implemented. An additional benefit of the exchange was the forging of strong relationships between both public advisors and researchers and the ongoing peer support.

**Consent to publish**

This research does not contain data from individual participants.

## P23 Developing a toolkit for Patient and Public Involvement in antimicrobial medicines development research: breaking new ground

### Michele Kok^1^, Andy Gibson^1^, David Evans^1^, Sally Grier^2^, Alasdair MacGowan^2^

#### ^1^Department of Health and Social Sciences, University of the West of England, Bristol, UK; ^2^Department of Medical Microbiology, North Bristol NHS Trust, Bristol, UK

##### **Correspondence:** Michele Kok (michele.kok@uwe.ac.uk)

**Introduction**

Patient and Public Involvement (PPI) in antimicrobial medicines development research is a new and exciting area. Involving patients/the public throughout the antimicrobial medicines development lifecycle can help ensure that research addresses their needs, help improve participation rates, and contribute to the successful dissemination of findings. However, there is currently no literature focusing on PPI in this area.

COMBACTE-MAGNET (www.combacte.com) is a consortium seeking new ways of treating multi- resistant bacterial infections. As part of its clinical coordinating work package, WP6I, we are developing a toolkit in collaboration with an acute infection and microbiology patient panel set up by the North Bristol NHS Trust (NBT) to provide evidence-based guidance for PPI in antimicrobial medicines development, including the role of PPI in setting the research agenda, clinical trials, and regulatory processes.

**Challenges and enablers of the toolkit development process**

PPI is relatively unheard of in the field of infectious disease and microbiology, with few established patient support groups or voluntary organisations in this area. Our newly established patient panel comprised people with experience of a serious infection requiring hospitalisation, but needed to be supported to enable them to effectively contribute to the toolkit development process. Initial meetings provided information about various topics related to antimicrobial resistance and medicines development, and included researcher-facilitated discussions. The quality of the panel’s contributions improved as their knowledge and confidence increased.

Our European partners within COMBACTE-MAGNET are less familiar with PPI in research. The role of PPI is often limited to marginal contributions due to a lack of understanding of where and how it fits into the research process. Our challenge was to convince them of the potential for and benefits of PPI in antimicrobial medicines development, in order to engage them in the toolkit development process. We organised workshops based on the toolkit content to enable them to explore the different roles of patient and public contributors throughout the antimicrobial medicines development lifecycle. Feedback from the workshops contributed to further development of the toolkit.

**Conclusion**

The toolkit is intended to change perception and increase receptivity of stakeholders towards PPI in antimicrobial medicines development research. Developing the toolkit with patient collaborators and European partners in an area of research that is driven by the pharmaceutical industry, with little/no experience of PPI to date has been challenging. Nevertheless, we have learned some key strategies that can facilitate the toolkit development process, and that can potentially be applied to other challenging acute clinical research areas.

**Acknowledgements**

This research project receives support from the Innovative Medicines Initiative (www.imi.europa.eu) Joint Undertaking under grant agreement n° 115523 | 115620 | 115737 resources of which are composed of financial contribution from the European Union Seventh Framework Programme (FP7/2007-2013) and EFPIA (European Federation of Pharmaceutical Industries and Association) companies in kind contribution. The authors acknowledge David Rowe and Richard Campbell for their contributions as representatives of the NBT acute infection and microbiology patient panel.

## P24 Walking the talk – Developing and modelling co-productive learning; the case for the Exchange Network

### Rachel Matthews^1^, Constantina Papoulias^2^, Cherelle Augustine^1^, Maurice Hoffman^1^, Mark Doughty^3^

#### ^1^NIHR CLAHRC Northwest London, Imperial College London/Chelsea and Westminster NHS Foundation Trust, London, UK; ^2^NIHR CLAHRC South London, King’s College, London, UK; ^3^The King’s Fund, London, UK

##### **Correspondence:** Rachel Matthews (r.matthews@imperial.ac.uk)

**Aim**

To tell the story of the co-design and testing of a shared learning space for patients, carers, researchers, managers and clinicians (the Exchange Network).

To demonstrate how that space works and share experiences from members.

**Background**

The network evolved through feedback from patient and carer advisers. It was deliberately co-designed to provide an engaging and supportive environment where people can connect, regardless of title, role or employment status, to share knowledge, skills and experience about improvement and research. It has a growing pool of 73 contacts who meet 4 times a year as varied groups of 15 to 20.

The network actively incorporates the INVOLVE values and principles into all aspects of its operation. It is designed to promote a space of respect, transparency and accountability; meetings are currently co-facilitated by a service-user and a manager who work together to model active and attentive listening. Particular issues are presented and open questions are encouraged; assumptions and judgments are challenged and real time feedback is provided. A quality improvement approach is used to adapt meetings, based on member suggestions.

**Impact**

Connected patients, carers, clinicians, managers and researchers

- Addressed power differentials between members to break down barriers and promote learning
- Tackled real world practical challenges (for example on implementing shared- decision making and working with established service user groups)
- Provided a space that fosters creative thought and active reflection – which cannot always be experienced in other settings

## P25 Developing good practice guidance for the involvement of public members in project oversight groups (Trial Steering Committees, Study Steering Groups)

### Heidi Surridge^1^, Doreen Tembo^1^, Amanda Roberts^2^, Eleni Chambers^3^

#### ^1^NIHR Evaluation Trials and Studies Coordinating Centre (NETSCC), Southampton, UK; ^2^Public member, NETSCC Public Involvement Virtual Network and Public member of a Trial Steering Committee, Southampton, UK; ^3^Public member, NETSCC PPI Reference Group, Southampton, UK

##### **Correspondence:** Heidi Surridge (Heidi.Surridge@nihr.ac.uk)

The aim of this poster is to present and build upon robust good practice guidance for involvement of public members in Trial Steering Committees (TSCs) and other project oversight groups.

Patient and Public Involvement (PPI) is embedded into the NIHR Evaluation Trials and Studies (NETS) Programmes’ management processes and research it funds. The current NETS Coordinating Centre (NETSCC) PPI Framework stipulates: “Studies that have a Trial Steering Committee or Study Steering Group must appoint a public member”. However, there was a lack of guidance on or evidence of the nature of public members’ contribution to work of oversight groups.

Therefore, Chairs and public members of NETS studies were interviewed to explore the role, value and impact of public members on study oversight groups. The results were used to draft good practice guidance and a role description.

This practical guidance will help Chairs of oversight groups, researchers and public members across NIHR and beyond, to recruit and facilitate effective and impactful public membership.

A group of Medical Research Council (MRC) methodology hub studies on oversight groups (specifically Trial Steering Committees) intend to develop new NIHR guidance and are sharing their public involvement findings as part of this development process.

The good practice guidelines will be presented highlighting the potential role of public members and opportunities will be provided for the audience to participate directly in their further development.

## P26 A personal reflection on being a co-applicant in the ACtiF study

### Daniel Beever^1^, Martin Wildman^2^

#### ^1^Clinical Trials Research Unit, School of Health and Related Research, University of Sheffield, Sheffield, South Yorkshire, UK; ^2^Sheffield Teaching Hospitals NHS Foundation Trust, Sheffield, South Yorkshire, UK

##### **Correspondence:** Daniel Beever (d.a.beever@sheffield.ac.uk)

**Background**

This poster aims to reflect on the involvement of Dan, a patient co-applicant and researcher, in the Adherence to treatment in adults with cystic fibrosis (ACtiF) study – a large programme grant. It is hoped this will be of interest to those considering involving a public co-applicant. It may also interest people thinking about becoming a co-applicant – particularly those with a research background.

ACtiF is looking at developing a support package to help people with cystic fibrosis (CF) take their treatments. Dan was invited to join the team as a person with CF (pwCF), though he also works as a research assistant. He also has experience of coordinating and facilitating public involvement work in various studies.

Despite Dan’s research background, he has found real freedom in focusing on only giving a patient perspective. As patient and public involvement (PPI) lead, Dan organises and leads the study’s PPI group teleconferences. He has been able to draw on his experience to publicise the opportunity to get involved.

As pwCF cannot meet due to cross-infection risk, the group has also given Dan the chance to talk about his health with other pwCF. This has included reflecting on many shared experiences. In addition, Dan also notes that he has got better at taking his treatments during his involvement with the study.

Dan’s experience shows that involvement of a patient co-applicant can be very positive, even beyond what is intended.

**Acknowledgements**

Presented on behalf of the ACtiF study group.

This abstract summarises independent research funded by the National Institute for Health Research (NIHR) under its Programme Grants for Applied Research Programme (Grant Reference Number RP-PG-1212-20015). The views expressed are those of the author(s) and not necessarily those of the NHS, the NIHR or the Department of Health.

## P27 Discovering the role of public co-applicant on a National Institute for Health Research (NIHR) Programme grant

### Rosemary L. Davies^1,2^ (rosemary3.davies@uwe.ac.uk)

#### ^1^Department of Health and Social Sciences, University of the West of England, Bristol, UK; ^2^National Institute for Health Research, Collaborations for Leadership in Applied Health Research and Care West (NIHR CLAHRC West), University Hospitals Bristol NHS Foundation Trust, Bristol, UK

**Background:**

Increasing numbers of members of the public are being appointed as co-applicants on National Institute for Health Research (NIHR) funded projects but there is no shared understanding of what is required in this role. The poster aims to share reflections on how the public co-applicant role developed within a five year programme grant focused on suicide prevention. This topic is important to both public contributors and researchers.

**Aims:**

- To share key reflections on the development of the role of service user advisor and co-applicant on an NIHR-funded Programme of suicide prevention research (2012-17), including working on a range of studies across three different universities.
- To share key issues for consideration when appointing public co-applicants in order to improve understanding of involvement of this kind.

**What difference has this project made:**

- Understanding how the role has contributed to the development of public involvement across the research programme.
- Including some of the impacts of involvement.
- Identifying key elements of the role.
- Sharing learning about working with this group of vulnerable people and some of the constraints for public involvement in suicide research.

**What will people learn from the poster:**

- Understanding of one interpretation of the co-applicant role.
- Key issues for reflection and consideration when planning similar involvement.

**Further information available about this work available from: Rosie Davies email: Rosemary3.Davies@uwe.ac.uk**

**Acknowledgements**

This work was funded by the National Institute for Health Research (NIHR) under its Programme Grants for Applied Research scheme (RP-PG-0610-10026).

## P29 Patient peer review in academic journals: developing guidance with *The BMJ* and *Research Involvement and Engagement*

### Sophie Staniszewska^1^, Richard Stephens^2^, Sara Schroter^3^, Amy Price^3,4^, Tessa Richards^3^, Andrew Demaine^5^, Rebecca Harmston^6^, Jim Elliot^7^, Ella Flemyng^8^

#### ^1^Warwick Research in Nursing, Warwick Medical School, University of Warwick, Coventry, UK; ^2^Involved and engaged patient and carer, Stevenage, UK; ^3^The BMJ, London, UK; ^4^Department of Continuing Education, University of Oxford, Oxford, UK; ^5^Involved and engaged patient, Saltash, UK; ^6^Involved and engaged patient, Norwich, UK; ^7^Health Research Authority, London, UK; ^8^BioMed Central, London, UK

##### **Correspondence:** Amy Price (aprice@bmj.com), Ella Flemyng (ella.flemyng@biomedcentral.com)

**Background**

Patient review embedded within journal processes is still new. *The BMJ* and *Research Involvement and Engagement (RIE)* were the first journals to routinely involve patient reviewers in their peer review processes. These two journals jointly carried out a survey of patient reviewers, developed with their patient reviewers, to ask them about their experience to help inform the development of comprehensive guidance for patient reviewers.

**Methods**

We surveyed patient reviewers who had recently reviewed for *The BMJ* or *RIE* to investigate their motivation to review, gather feedback on their experience of patient review and the support available, and to find out how the process could be improved. We collated the results and identified emerging themes to inform the development of comprehensive, evidence-based patient review guidance, to help ensure patient reviewers feel supported when conducting patient review. This will create a basis for other journals wanting to implement and systematically integrate patient review in their processes.

**Call to action**

We report our key results in this poster and call for attendees at the INVOLVE conference to reflect on the findings. We invite readers of this poster to leave feedback or comments in an envelope attached to the poster, and thus to join this world-first project involving patients (and others) in developing guidance for patients reviewing submissions for academic journals.

**Consent to publish**

This abstract does not contain any identifiable information of individual participants and therefore doesn’t require consent for its publication.

**Competing interests**

Sophie Staniszewska and Richard Stephens are co-Editors-in-Chief of *Research Involvement and Engagement*. Jim Elliot is an Editorial Board Member and reviewer for *Research Involvement and Engagement.* Andrew Demaine and Rebecca Harmston are patient reviewers for *The BMJ*. Ella Flemyng is employed by BioMed Central. Sara Schroter is employed by *The BMJ*. Amy Price is a research fellow at *The BMJ* and a member of the BMJ’s Patient Advisory Pane

## P30 Patient involvement at every stage: design and coproduction of the Head Up neck support collar

### Lise Sproson^1^, Liz Pryde^1^, Heath Reed^2^, Gill Squire^3^, Andy Stanton^2^, Joe Langley^2^, Moya Briggs^1^, Philip Brindle^1^, Rod Sanders^1^, Christopher McDermott^3^

#### ^1^NIHR Devices for Dignity Health Technology Co-operative, Sheffield, UK; ^2^Lab4Living, Art and Design Research Centre, Sheffield Hallam University, Sheffield, UK; ^3^Sheffield Institute for Translational Neuroscience, University of Sheffield, Sheffield, UK

**Background**

Many neurological conditions, including myasthenia gravis, spinal muscular atrophy and motor neurone disease/amyotrophic lateral sclerosis cause neck weakness.^1^ Patients who experience this have difficulty holding up their head, resulting in pain, and problems with eye contact, communication, use of computers and television and loss of confidence in going out^2^.

The need for this project came directly from users and carers. The project was initially proposed by a Clinical Studies Group for Motor Neuron Disease (MND), who approached NIHR Devices for Dignity (D4D) with the view that the current cervical orthoses are inadequate in terms of function and comfort. Soft collars were comfortable against the skin, but provided insufficient support whereas more rigid collars provided head support but restricted movement and produced skin soreness.

**Methods**

The project was multi-disciplinary, involving expert patients, researchers, clinicians, academics and designers. The patient experts explained the impact of neck weakness on their life and limitations of existing supports and also suggested what design requirements would be necessary in order to develop a new neck orthosis that was flexible and comfortable yet supportive.

The project utilised a co-design process, meeting regularly to evaluate designs and prototypes. Once consensus was reached, 150 Head Up collars were manufactured for evaluation by patients and clinicians in the Head Up study.

Social media was harnessed to disseminate the patient voice throughout all project stages - examples include YouTube patient interview videos, patient blogs, Twitter and Facebook groups.

**Results**

140 patients were recruited from 10 centres across the UK and Ireland. 116 patients completed the study, and of these, 80% chose to keep the Head Up collar and continue to use it in preference to other collars after the month trial period. Head Up scored significantly better (p<0.005) than previous collars used by patients in terms of satisfaction, level of support offered, residual head movement possible, appearance, lack of interference with eating and drinking.

We look forward to disseminating the results of the full study in the near future and making the Head Up collar available to all patients who might benefit.

**Conclusions**

We have learned much from our co-production journey and feel that the Head Up collar could only be fit for purpose via working in this way. The journey has been more powerful than any of us might have imagined at the start^3^.

**Acknowledgements**

This research was funded by the National Institute for Health Research (NIHR) Invention for Innovation (i4i) programme II-ES-0511-21003. The views expressed are those of the author(s) and not necessarily those of the NHS, the NIHR or the Department of Health.

The MND Association care centre network recruited volunteers to join the design process and also assisted in recruiting to the evaluation phase. We would like to thank the patients and their carers who volunteered to try the new orthosis, and who gave up their valuable time to provide us with their assessments and detailed feedback.

**The authors above reflect project team members involved in production of this poster, (for space restriction reasons). We wish in addition to acknowledge other members of the main project team:** Mrs Zoe Clarke – Barnsley Hospital NHS Foundation Trust, Dr Nicola Heron – NIHR Devices for Dignity hosted by Sheffield Teaching Hospitals NHS Foundation Trust, Mrs Ann Quinn – South Yorkshire MND Association, (Dame) Professor Pamela Shaw – University of Sheffield, Susan Baxter – University of Sheffield, Simon Judge – Barnsley Hospital NHS Foundation Trust, Dr Avril McCarthy – NIHR Devices for Dignity hosted by Sheffield Teaching Hospitals NHS Foundation Trust, Oliver Wells – NIHR Devices for Dignity hosted by Sheffield Teaching Hospitals NHS Foundation Trust, Mark Strong – University of Sheffield, Thomas Bartlett, University of Sheffield and Professor Wendy Tindale – NIHR Devices for Dignity hosted by Sheffield Teaching Hospitals NHS Foundation Trust.

**References**

1. Gourie-Devi J, Nalini A, Sandhya S. Early or late appearance of ‘dropped head syndrome’ in amyotrophic lateral sclerosis. J Neurol Neurosurg Psychiatry. 2003;74:683–6.

2. Glazener P. Pilot study to determine the effectiveness of a new neck brace design for patients with amyotrophic lateral sclerosis. J Nurs Educ Pract. 2014:4:1–5.

3. Heath, R. et al. (2015). Head up: an interdisciplinary, participatory and co-design process informing the development of a novel neck support for people living with progressive neck muscle weakness. Journal of Medical Engineering & Technology, 39 (7), 404-410.

Logos, links to social media and images will be included in the poster.

## O7 The benefit of a partnership approach enabling the patient voice to be heard loud and clear and the added value this brings to the research

### Coyle David^1^, Heron Nicola^1^, Davies Simon^2,3^, Wilkie Martin^3^

#### ^1^NIHR Devices for Dignity Healthcare Technology Co-operative at Sheffield Teaching Hospitals NHS Foundation Trust, Royal Hallamshire Hospital, Glossop Road, Sheffield, S10 2JF, UK; ^2^Institute for Applied Clinical Sciences, Keele University, Keele, Staffordshire, UK; ^3^University Hospital of North Midlands, Newcastle Rd, Stoke-on-Trent, Staffordshire, ST46QG, UK; ^4^Renal Medicine, Sheffield Teaching Hospitals NHS Foundation Trust, Sheffield, UK

**Correspondence:** Coyle David

Our aim is to share the impact patients as co-applicants can have on research through describing the breadth and depth of the involvement.

Devices for Dignity (D4D) is a National Institute for Health Research (NIHR) funded Healthcare Technology Co-operative. This work describes how D4D supported the patient and public involvement in the development and delivery of an NIHR Health Technology Assessment grant.

We will cover the follow stages of the research process:

1. Early Involvement

- We will describe the process for involvement at the grant development stage and the impact that had on the final application.

2. Role and remit for patients as partners We will:

- Describe how the programme was designed to ensure patient involvement was integral throughout; including the core research and project oversight groups
- Explain the critical roles for patient co-apps in recruitment and retention and in developing a communication and dissemination plan
- Discuss how we are working closely with the project charity partners.
- Describe our consideration of types of patient knowledge, experience and skills needed on a Patient Advisory Group

This presentation is important to any research team looking at the role of a patient as a co- applicant. It demonstrates the value that this can bring if the patient is viewed as an essential and equal partner in the research.We will demonstrate the process for reaching this level of patient partnership and hope to influence and support further research teams and members of the public to adopt this approach.

## O14 Working together to advance public involvement: Coproduction and partnership working within a regional public involvement network

### Tina Coldham^1^, Claire Ballinger^1^, Lynn Kerridge^2^, Mark Mullee^3^, Caroline Eyles^3^, Megan Barlow-Pay^3^

#### ^1^Wessex PIN, Southampton, UK; ^2^NETSCC, University of Southampton, Southampton, UK; ^3^Research Design Service South Central, Southampton General Hospital, Southampton, UK

##### **Correspondence:** Tina Coldham (TRColdham@btinternet.com)

**Aims of the session**

In this workshop, we will:

- Communicate the vision and our personal motivations (lay and professional) for the Wessex Public Involvement Network (PIN)
- Explore with participants principles and practicalities in establishing a regional PIN
- Together identify generalisable facilitators and barriers to the implementation of regional PINs

**Why is it important, and to whom?**

This workshop will be of interest and importance to all who facilitate, contribute to and use outcomes from public involvement (PI) in health research: patients, the public, PI staff leads, health care staff and researchers. We will demonstrate ways of working together, promoting best practice and taking forward leadership in PI with both lay and staff contributors.

**What difference has, or could this project make?**

We will share the achievements of the Wessex PIN to date, including: partnership working across regional NIHR and NHS organisations; a jointly organised and facilitated community PI event; ongoing opportunities for shared learning, support and reflection.

With workshop participants, we will explore potential longer term benefits of regional PINs including: easier and fairer access to PI opportunities; economies of scale; and learning and career trajectories for PI leads.

**What will people take away from this session?**

Those participating in this workshop will:

- Explore a real-life example of co-production and partnership working in a regional PIN
- Reflect on opportunities and challenges which the Wessex PIN model offers within their own working context
- Identify benefits, resources and contacts to support similar regional PINs

**CONFERENCE THEME(S)**

Regional Networks

## O18 Developing principles for co-producing health and social care research

### Gary Hickey^1^, Tracey Johns^2*^, Jon Paylor^3^, Katie Turner^4^

#### ^1^NIHR INVOLVE, University of Southampton, Southampton, UK; ^2^NIHR Research Design Service East of England, University of Essex, Essex, UK; ^3^NIHR Research Design Service London, Kings College London, London, UK; ^4^Population Health Research Institute, St George’s, University of London, London, UK

##### **Correspondence:** Tracey Johns (tracey.johns@essex.ac.uk)

**Background**

In recent years we have seen a growing interest in applying the concept of co-production in the field of health and social care research. There is, however, much variation in the definition and practice of co-production, revealing a lack of clarity around the concept. Co- production, it has been suggested (Going the Extra Mile 2015), could be a means of evolving and improving patient and public involvement in research. But co-production can be a slippery concept, reflecting the wide range of disciplines from which it emerges and the frequently loose way it is applied. Moreover patient and public involvement in research already has its own vocabulary. So what is co-production and what does it mean for patient and public involvement in research? Co-producing research is an emerging field challenging how we think about and do research and the relationships between organisations, professionals and researchers and the public.

**Aim**

To help NIHR/INVOLVE identify some key principles and features involved in co-producing research. And to develop guidance to support organisations, researchers and the public to evaluate their own (and others) practices and further evolve and improve public involvement in their research.

**Approach**

The draft guidance draws on findings from a round table meeting held to discuss ‘co- producing research’, a literature review and interviews with people involved in co-produced research (undertaken by Jonathon Paylor, RDS London and Tracey Johns, RDS East of England), a workshop to gain consensus on the key principles and elements of co-producing research and consultation with NIHR staff and beyond.

The principles included are just the beginning of a pathway for those considering taking a journey on the co-production route. The extent to which research projects and organisations embrace all of the principles and the depth to which they go in embedding the principles will vary. The more principles that are adopted and embedded the stronger will be the co- production of the research. There is no single formula for co-production and such an approach would be counter to the innovation and flexibility that is implicit in co-produced research.

**Acknowledgements**

Thank you to those who took part in the interviews and also the members of the NIHR INVOLVE co-production task group [http://www.invo.org.uk/current-work/co-production/].

## *P4 “We have a voice”* [Matt]

### Lisa Whiting, Sheila Roberts, Julia Petty, Gary Meager

#### Department of Nursing and Social Work, University of Hertfordshire, Hatfield, Hertfordshire, England

##### **Correspondence:** Lisa Whiting (L.Whiting@herts.ac.uk)

**Aim:**

The poster presents the evaluative mixed methods research study that was commissioned by NHS England and undertaken by the University of Hertfordshire, July 2015 - September 2016. The study examined the role of the NHS England Youth Forum [NHSEYF] members as well as the strategies they undertook to influence health service provision for children and young people [CYP].

**Why is the research important?**

The NHSEYF is a unique model that others are seeking to emulate; it was therefore imperative that its role and influence was evaluated. The researchers involved members of the NHSEYF (young people aged 14-25 years) by holding meetings with them at their residential weekends to discuss and plan the stages of the research.

The study comprised of quantitative data collection via ‘Activity Logs’; these were completed (over a three month period) by nine of the NHSEYF; semi-structured interviews were then undertaken with eight NHSEYF members.

**What difference has the research made?**

Findings revealed that the NHSEYF members are:

- Undertaking an enormous range of activities;
- Positively influencing healthcare provision for CYP;
- Extremely capable of being involved in decision-making;
- Totally committed to ensuring CYPs’ voices are heard;
- Inspiring other CYP to be involved in health-related organisations (e.g. local Youth Forums).

**What can people take away?**

The Youth Forum Wheel (see image) that depicts the *‘components of success’* underpinning the NHSEYF; this has the potential to underpin the development and operationalisation of other youth forums, both within, and outside of, a health context.
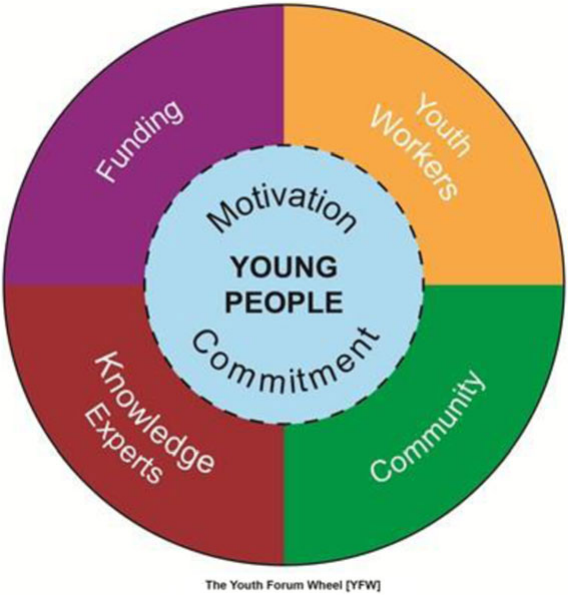


**Acknowledgements**

The research team would like to gratefully acknowledge:

- NHS England for commissioning and funding the project.
- All of the young people who took part in the study and who gave their time so willingly.

## P5 Alzheimer’s Society Research Network: the impact of involving people affected by dementia in research

### Anna Grinbergs-Saull, Natasha Morgan

#### Alzheimer’s Society, London, UK

##### **Correspondence:** Anna Grinbergs-Saull

Established in 1999, the Alzheimer’s Society Research Network now involves over 280 people with dementia, carers and former carers. The Research Network is involved in every stage of our research programme, from reviewing all applications for funding, to monitoring and delivering research we fund and support. Drawing on 18 years of experience, we will present the impact that the Research Network has had and continues to have on the research and people we work with. Using case studies developed with Research Network volunteers and dementia researchers, we will show the impact of Patient and Public Involvement in four key areas. These are:

- impact on research
- impact on researchers
- impact on the people affected by dementia who are involved
- impact on Alzheimer’s Society as an organisation.

We will share what we have learnt as a Network, and our plans for further development; discussing how to create and maintain a culture of involvement in a research funding charity.

## P19 Coproduction in action: The work of the Peer Expertise in Education and Research (PEER) Group

### Kati Turner, Flavia Collins, Sarah Gibson, Siobhan Passmore

#### Population Health Research Institute, St George's, University of London, London, UK

##### **Correspondence:** Kati Turner

**Background**

- The Peer Expertise in Education and Research (PEER) Group is made up of service users and carers belonging to South West London & St George's Mental Health NHS Trust (SWLStG).
- It is the primary resource for public and patient involvement (PPI) in research and education for clinicans and researchers from SWLStG and St George's, University of London (SGUL).
- The group has been cited in INVOLVE publications as an example of ethical and principled PPI practice. It is co-facilitated by two service user researchers.

**Aims**

- Explaining the purpose, activities and outcomes of the group.
- Passing on the learning and experience of group members and facilitators regarding working coproductively around involvement in research.

**Importance**

- Demonstrates - to academic, clinical and service user researchers, members of the public and PPI leads - the value and importance of PPI within mental health Trust and university settings.
- Models good practice, values and ethos in line with those of INVOLVE.
- Opportunity to talk to group members directly about their experiences.

**Impact**

- Our poster will demonstrate the impact of successful coproductive ways of working in PPI in research in terms of:
  - ◦ Process – the way group members work together.
  - ◦ Outcomes – the impact on research proposals and projects.

**What will people take away**

- Knowledge of what makes coproduction in PPI work well and what the challenges are.
- What is needed in terms of support and training for coproduction in PPI to work well.

**Acknowledgements**

Production of this poster was led by a small working group of PEER Group members following input from all group members.

## P21 Using the 4Pi National Involvement Standards as a framework to engage service users/patients: A quality improvement perspective

### Liz Evans^1^, Stuart A. Green^1^, Jenny Trite^2^, Rachel Matthews^1^

#### ^1^NIHR CLAHRC Northwest London, Imperial College London/Chelsea and Westminster NHS Foundation Trust, London, UK; ^2^Central and Northwest London NHS Foundation Trust, London, UK

##### **Correspondence:** Stuart A. Green (stuart.green@imperial.ac.uk)

**Aim**

This session aims to provide a practical guide to the use of the 4PI National Involvement Standards as part of a quality improvement initiative.

**Background**

National and local policy supports the involvement of patients at all levels in the design, delivery and improvement of health services. Quality improvement methods and approaches, such as tests of change, are commonly used to improve the delivery of care and health outcomes, and should include the involvement of patients and the public. CLAHRC Northwest London, an applied health research programme funded by the NIHR, used the 4Pi National Involvement Standards as a guiding framework for involving service users/patients in quality improvement initiatives.

**Impact**

Including the 4Pi standards within a systematic approach to quality improvement in NHS organisations can facilitate the involvement of service users/patients and provide an effective mechanism to introduce changes in clinical care. The framework also offers those involved in quality improvement a structured approach to ensure that involvement is meaningful and transparent.

**Lessons learned**

The application of the 4Pi framework promotes the creation of meetings accessible for all team members that encourages active participation, which also extends the benefits beyond the service users/patients to flatten hierarchies within the wider improvement team. In addition its use clearly demonstrates the benefits of working with service users/patients at an organisational and clinical level that encourages support from senior leaders and healthcare professionals. Despite this, organisational constraints still exist and should be recognized and dealt with to encourage the inclusion and full participation of service users/patients.

## P22 Integrating Perspectives on Evaluation of Patient & Public Involvement in Research

### Susan Hrisos^1^, Richard Thomson^1^, Dave Green^1^, Helen Atkinson^2^, Alex Mitchell^2^, Lynne Corner^2^

#### ^1^Institute of Health & Society, Newcastle University, Newcastle, UK; ^2^Faculty of Medical Sciences Engage, Newcastle University, Newcastle, UK

##### **Correspondence:** Susan Hrisos (susan.hrisos@ncl.ac.uk)

Patient and Public Involvement (PPI) has developed substantially in the last decade. Key drivers of this progress include iterative policy developments, an accumulating body of practice examples, the availability of practical guidance, and common-ground understanding of the values and principles that underpin good practice. Evaluation of PPI is considered important to inform continuous improvement in practice, to inform policy and strengthen the evidence-base, and to maximise impact. Understanding how, what and when to evaluate can be difficult as there are several different perspectives on this.

Addressing this through wide consultation is important in order to inform meaningful evaluation and impactful involvement. This poster aims to prompt debate on the topic of evaluation of PPI in research. As well as the findings from a literature review by the authors, the poster will reflect on the outcomes of our recent national consultation with UK-based experts, which considered issues from a range of viewpoints. To help shape the future direction of evaluation of PPI in a way that is knowledgeable of, informed by and considerate of all perspectives, the authors wish to build on and broaden this discussion to a wider public. The poster will therefore engage delegates with key questions, and invite their thoughts on the issues raised, in conversation with the authors and /or via post-it notes attached to the poster board. The poster will also present information about a future consultation event with PPI contributors, and a planned international conference on the same topic.

## P31 Using innovative approaches to involve young people in health research

### Anne McKenzie AM^1^ (Anne.Mckenzie@uwa.edu.au), Rebecca Nguyen^1,2^ (Rebecca.Nguyen@uwa.edu.au), Belinda Frank^1^ (Belinda.Frank@uwa.edu.au), Ngaire McNeil^1^ (Ngaire.Mcneil@uwa.edu.au), Hayley Harrison^1^ (Hayley.Harrison@uwa.edu.au)

#### ^1^Consumer and Community Health Research Network, Perth, Australia; ^2^Telethon Kids Institute, Perth, Australia

**Background**

The value of the youth voice informing research priorities, policy and practice is increasingly being recognised across the globe. Not only does youth involvement benefit the organisation, young people also gain more skills, build a sense of empowerment and establish new networks ^1^.

Until recently, the research agenda at the Telethon Kids Institute (the ‘Institute’) in Perth, Western Australia, was missing input from this key group

A planned strategy developed by the Consumer and Community Health Research Network (the Network) was instrumental in the eventual outcome of establishing an active 23-member Youth Advisory Group at the Institute. This involvement initiative has empowered young people and informed researchers of issues facing young people not previously considered.

**Materials and Methods**

The strategy to incorporate the youth voice included holding a community forum with 40 young people aged 14 – 25 years old, to discuss their preferred method for establishing the group. The Youth Advisory Group was established following a recruitment drive via the Network ‘s community database. The first meeting was held in 2016 and used a World Café methodology to encourage a relaxed and comfortable atmosphere that recognises individual and cultural preferences in communication^2^.

Facebook is currently used as: a platform for discussion of research projects; and communicating meeting and event planning. Mentimeter, an interactive online polling system, is used for members to anonymously vote on issues with their personal smartphones at meetings.

**Results**

The Youth Advisory Group played a key role in developing the Institute’s ‘Think Big’ strategic research initiative. Members completed an online survey and attended a priority-setting workshop to discuss and develop a list of ‘Big Ideas’ for future research.

The formation of the Institute’s Youth Advisory Group has also lead to young people having input into the Western Australian Youth Health Policy. In collaboration with WA Health Department six community forums have been held across Western Australia. The Community Conversations have been an outstanding success. The Youth Advisory Group provided input into the forum’s questions, attended forums and helped to promote the events through their networks.

**Conclusions**

It is vital that the youth community voice is heard in research organisations that directly relate to children and young people’s health. Involving young people has many benefits including

- Addressing gaps in research that are of importance to young people
- Ensuring the research is relevant and informed by this group of the population

**References:**

1 http://mypeer.org.au/design-implementation/youth-participation-2/benefits-of-youth-participation/

2 http://www.theworldcafe.com/method.html

