# Correction to: Abstracts from the NIHR INVOLVE Conference 2017

**DOI:** 10.1186/s40900-018-0090-6

**Published:** 2018-02-22

**Authors:** Elspeth Mathie, Helena Wythe, Diane Munday, Paul Millac, Graham Rhodes, Nick Roberts, Jean Simpson, Nat Barden, Penny Vicary, Amander Wellings, Fiona Poland, Julia Jones

**Affiliations:** 1 CRIPACC, University of Hertfordshire, Hatfield, Hertfordshire UK; 2 Public Involvement in Research Group, University of Hertfordshire, Hatfield, Hertfordshire UK; 3 INsPIRE PPI Group, Cambridgeshire Community Services NHS Trust, Ely, Cambridgeshire UK; 4 Cambridge University Hospital (CUH) Patient and Public Involvement Panel, Cambridgeshire, UK; 5 Service User and Research Group, Cambridge and Peterborough Foundation Trust, Cambridgeshire, UK; 6 Public & Patient Involvement in Research (PPIRes), Norfolk and Suffolk, UK; 7 University of East Anglia, Norwich, Norfolk UK

## Correction

After publication of this supplement [1] it has come to our attention that in abstract O13 Regional working in east of England: co-designing a PPI feedback tool the funding and disclaimer statement were omitted. It should have instead included these statements. The NIHR funding and disclaimer statements appear below.

NIHR funding statement:

This is a summary of independent research funded by the National Institute for Health Research (NIHR) Collaborations for Leadership in Applied Health Research and Care East of England (CLAHRC EoE) Programme.

NIHR disclaimer statement:

The views expressed are those of the author(s) and not necessarily those of the NHS, the NIHR or the Department of Health.

This has now been included in this erratum.

**O13 Regional working in east of England: co-designing a PPI feedback tool**

Elspeth Mathie^1^, Helena Wythe^1^, Diane Munday^2^, Paul Millac^2^, Graham Rhodes^3^, Nick Roberts^3^, Jean Simpson^4^, Nat Barden^5^, Penny Vicary^6^, Amander Wellings^6^, Fiona Poland^7^, Julia Jones^1^

^1^CRIPACC, University of Hertfordshire, Hatfield, Hertfordshire, UK; ^2^Public Involvement in Research Group, University of Hertfordshire, Hatfield, Hertfordshire, UK; ^3^INsPIRE PPI Group, Cambridgeshire Community Services NHS Trust, Ely, Cambridgeshire, UK; ^4^Cambridge University Hospital (CUH) Patient and Public Involvement Panel, Cambridgeshire, UK; ^5^Service User and Research Group, Cambridge and Peterborough Foundation Trust, Cambridgeshire, UK; ^6^Public & Patient Involvement in Research (PPIRes), Norfolk and Suffolk, UK; ^7^University of East Anglia, Norwich, Norfolk, UK

**Correspondence**: Elspeth Mathie (e.j.mathie@herts.ac.uk)

Research Involvement and Engagement 2017, **3(Suppl 1**):O13

**Background**

The importance of feedback is highlighted in the ‘Values and Principles’ [1] from INVOLVE and included in the current Public Involvement consultation on standards [2]. Patient and Public Involvement (PPI) contributors in the East of England (EoE) regional network flagged up the issue that feedback (from researchers to PPI contributors) was minimal or absent, so we co-designed a study to look at this. PPI contributors talked of spending valuable time commenting on complex issues and continue to volunteer without acknowledgement and thanks. The study aims to improve PPI feedback by codesigning a generic PPI Feedback process which can be adapted for individual PPI groups and activities.

**Methods**

The six regional PPI groups involved in the study include those based within the Research Design Service, Universities, hospitals and NHS Trusts. The study used a survey, interviews and 4 month audit. Over 100 respondents completed the survey distributed by the PPI groups and 23 PPI contributors, researchers and PPI leads were interviewed. Following two stakeholder meetings with researchers, PPI representatives and PPI group leads, local feedback tools were co-designed, implemented and trialled in the PPI groups. A second audit was undertaken by PPI representatives and PPI group leads to ascertain whether satisfaction with feedback had improved. Work is ongoing to identify barriers and facilitators to implementing the local tools and to co-develop the local tools to form a single regional EoE tool or process.

**Results**

The results confirmed the anecdotal evidence; feedback is not routine and very variable. Together, our research team (PPI contributors, leads, researchers) will outline our motivations for this research approach and our Feedback Tools. We will also discuss our results on the variation and frequency of feedback, barriers and enablers.

**Conclusion**

We aim to encourage other PPI groups to work together to improve feedback whilst underlining the importance of managing expectations and simultaneously nurturing relationships. A regional PPI Feedback tool or process is in development which we aim to produce and distribute in different user-formats.

**Acknowledgements**

Study stakeholder and research group; PPI group Leads and PPI groups.

**NIHR funding statement:**

This is a summary of independent research funded by the National Institute for Health Research (NIHR) Collaborations for Leadership in Applied Health Research and Care East of England (CLAHRC EoE) Programme.

**NIHR disclaimer statement:**

The views expressed are those of the author(s) and not necessarily those of the NHS, the NIHR or the Department of Health.

**References**

1. INVOLVE. Public involvement in research: values and principles framework. INVOLVE; Eastleigh. 2015.

2. https://sites.google.com/nihr.ac.uk/pi-standards/home

**P13 Embedding patient and public involvement (PPI) in a regional research network and beyond: findings and action points from the IMPRESS project and Collaboration for Leadership in Applied Health Research and Care (CLAHRC) East of England**

Julia Keenan^1^, Fiona Poland^1^, Helena Wythe^2^, Amander Wellings^3^, Penny Vicary^3^

^1^University of East Anglia, Norwich, Norfolk, UK

^2^University of Hertfordshire, Hatfield, Hertfordshire, UK

^3^Members of the Patient and Public in Research Group (PPIRes), NHS South Norfolk Clinical

Commissioning Group, Norwich, Norfolk, UK

^*^Corresponding Author: Helena Wythe (h.f.wythe@herts.ac.uk)

**Correction**

After publication of this supplement [1] it has come to our attention that in abstract “P13 Embedding patient and public involvement (PPI) in a regional research network and beyond: findings and action points from the IMPRESS project and Collaboration for Leadership in Applied Health Research and Care (CLAHRC) East of England” the funding and disclaimer statement were omitted. It should have instead included these statements. The NIHR funding and disclaimer statements appear below.

NIHR funding statement:

This is a summary of independent research funded by the National Institute for Health Research (NIHR) Collaborations for Leadership in Applied Health Research and Care East of England (CLAHRC EoE) Programme.

NIHR disclaimer statement:

The views expressed are those of the author(s) and not necessarily those of the NHS, the NIHR or the Department of Health.

This has now been included in this erratum.

**P13 Embedding patient and public involvement (PPI) in a regional research network and beyond: findings and action points from the IMPRESS project and Collaboration for Leadership in Applied Health Research and Care (CLAHRC) East of England**

Julia Keenan^1^, Fiona Poland^1^, Helena Wythe^2^, Amander Wellings^3^, Penny Vicary^3^

^1^University of East Anglia, Norwich, Norfolk, UK; ^2^University of Hertfordshire, Hatfield, Hertfordshire, UK; ^3^Members of the Patient and Public in Research Group (PPIRes), NHS South Norfolk Clinical Commissioning Group, Norwich, Norfolk, UK

**Correspondence:** Fiona Poland (f.poland@herts.ac.uk)

Research Involvement and Engagement 2017, 3(Suppl 1):P13

**Background**

We share findings from an action research project (IMPRESS: Implementing PPI in an NHS Research Programme: Evaluating the PPI contribution to CLAHRC research implementation) which studied how PPI has been implemented within a regional, applied research programme (a CLAHRC: Collaboration for Leadership in Applied Health Research and Care). This builds on findings from a previous national study (RAPPORT: ReseArch with Patient and Public invOlvement: a RealisT evaluation). Our project team includes two PPI coresearchers and an advisory group with a lay chair and further PPI representatives. IMPRESS employed a theoretical framework to explore in-depth, the experiences of PPI within the CLAHRC programme, from different points of view. Our findings identified the barriers and facilitators to the programme’s aim of ‘fully embedded, active and comprehensive’ PPI which then inform ten key action points for developing PPI in a programme. The network of CLAHRCs are planned to play a key role in codeveloping and co-delivering NIHR’s PPI strategy across regions in England. The CLAHRC studied here makes policy and resource commitments to PPI, has PPI as a research theme and works in partnership with regional PPI networks. It is thus important to report systematically researched findings on processes and outcomes of this commitment, both to inform specific local action and to report broader conceptual lessons for PPI knowledge and practice. We detail, with illustrative examples, how 10 case study projects made sense of PPI, bought into PPI, enacted PPI and appraised PPI. The action research approach enables, actions and solutions to problems of embedding PPI to be ‘fine-tuned’ in further research cycles to evidence and enact sustainable PPI processes and outcomes for all stakeholders. See a film of the study results at: https://www.youtube.com/watch?v=sL9EbvYmaxA

**Acknowledgements** Wider IMPRESS team members: Amanda Howe, Jonathan Boote, Anna Varley, study advisory group members.

**NIHR funding statement:**

This is a summary of independent research funded by the National Institute for Health Research (NIHR) Collaborations for Leadership in Applied Health Research and Care East of England (CLAHRC EoE) Programme.

**NIHR disclaimer statement:**

The views expressed are those of the author(s) and not necessarily those of the NHS, the NIHR or the Department of Health.
